# Transcriptome analysis of the filamentous fungus Aspergillus nidulans directed to the global identification of promoters

**DOI:** 10.1186/1471-2164-14-847

**Published:** 2013-12-03

**Authors:** Christopher Sibthorp, Huihai Wu, Gwendolyn Cowley, Prudence W H Wong, Paulius Palaima, Igor Y Morozov, Gareth D Weedall, Mark X Caddick

**Affiliations:** Institute of Integrative Biology, University of Liverpool, Biosciences Building, Crown Street, Liverpool, L69 7ZB UK; Department of Computer Science, University of Liverpool, Ashton Building, Ashton Street, Liverpool, L69 3BX UK; Department of Biomolecular and Sports Sciences, Faculty of Health and Life Sciences, Coventry University, James Starley Building, Coventry, CV1 5FB UK

**Keywords:** *Aspergillus nidulans*, Transcriptome, RNA-seq, Gene annotation, Alternative splicing, Natural antisense transcripts, Transcription start sites, Transcription factor binding sites

## Abstract

**Background:**

The filamentous fungus *Aspergillus nidulans* has been a tractable model organism for cell biology and genetics for over 60 years. It is among a large number of Aspergilli whose genomes have been sequenced since 2005, including medically and industrially important species. In order to advance our knowledge of its biology and increase its utility as a genetic model by improving gene annotation we sequenced the transcriptome of *A. nidulans* with a focus on 5′ end analysis.

**Results:**

Strand-specific whole transcriptome sequencing showed that 80-95% of annotated genes appear to be expressed across the conditions tested. We estimate that the total gene number should be increased by approximately 1000, to 11,800. With respect to splicing 8.3% of genes had multiple alternative transcripts, but alternative splicing by exon-skipping was very rare. 75% of annotated genes showed some level of antisense transcription and for one gene, *meaB*, we demonstrated the antisense transcript has a regulatory role. Specific sequencing of the 5’ ends of transcripts was used for genome wide mapping of transcription start sites, allowing us to interrogate over 7000 promoters and 5′ untranslated regions.

**Conclusions:**

Our data has revealed the complexity of the *A. nidulans* transcriptome and contributed to improved genome annotation. The data can be viewed on the AspGD genome browser.

**Electronic supplementary material:**

The online version of this article (doi:10.1186/1471-2164-14-847) contains supplementary material, which is available to authorized users.

## Background

The filamentous fungus *Aspergillus nidulans* is a model organism for many aspects of cell biology and genetics. Additionally, the aspergilli themselves include fungi of biomedical, agricultural and industrial significance [1–5]. Thus it is important to extend our understanding of *A. nidulans* to facilitate analysis of key processes which underpin fungal pathogenicity and biotechnological applications. The *A. nidulans* genome was sequenced and annotated in 2005 [6] and the genome annotation has been updated several times since then. It is among a relatively large number of sequenced *Aspergillus* genomes, genomic data for which are accessible via a number of public web resources [7–10]. Despite this wealth of data and manual curation efforts [11], genome annotations are largely based upon computational gene model predictions which may be inaccurate, a particular problem for complex genes containing multiple introns.

New sequencing technologies allow high-throughput sequencing of transcripts (RNA-seq) [12]. These data can be used to detect differential gene expression among different populations of cells, verify or correct gene models and identify previously unannotated transcribed regions of the genome. These include genes bound for translation as well as untranslated (non-coding) transcripts (ncRNA) that play important functional roles. Families of ncRNA include ‘housekeeping’ molecules such as transfer RNA (tRNA), ribosomal RNA (rRNA), spliceosomal RNA and small nuclear and small nucleolar RNA (snRNA and snoRNA). They also include ncRNA with putative regulatory roles such as small interfering RNA (siRNA), micro RNA (miRNA) and long ncRNA. The latter group include antisense transcripts which may be common in eukaryote transcriptomes [13], potentially playing roles in transcriptional and post-transcriptional regulation [14]. RNA-Seq has been applied to several *Aspergillus* species to show transcriptional responses to: biofilm growth in *A. fumigatus*[15]; growth on lignocellulose and germination of conidia in *A. niger*[16–18]; and temperature changes and 5-azacytidine in *A. flavus*[19, 20]. These and other studies [21, 22] have also explored *Aspergillus* transcriptomes more generally and improved genome annotation.

Knowledge of the precise position of a transcript’s 5′ end provides valuable data for gene annotation by defining the transcription start site (TSS) and thereby both the 5′ untranslated region (UTR) and upstream promoter region of the respective gene. This allows the identification of functionally important sequence elements that may then be analysed directly or *in silico*. However, the 5′ end of a transcript is difficult to distinguish accurately from whole transcriptome RNA-seq data due to uneven read coverage and bias in libraries leading to a low frequency of sequence reads from transcript ends [23]. Several methods to define the 5′ ends of transcripts have been described, using different sequencing technologies and with varying levels of throughput [24–27].

Here, we describe both the strand specific sequencing of the whole transcriptome of *A. nidulans* and the specific sequencing of transcript 5′ ends. The latter was achieved by adapting 5′ RATE (‘robust analysis of 5′-transcript ends [26]). By varying the pre-treatment of RNA we were able to specifically sequence capped, uncapped or both capped and uncapped transcripts. The sequencing of transcript 5’ ends led to the identification of over 7000 transcription start sites across the genome and these were used to investigate common features associated with transcription sites and promoters, including the identification of novel motifs. We found many cases of antisense transcription of protein coding genes (up to 72% of genes), as well as modest levels of alternative splicing of transcripts (up to 8.2% of genes). The occurrence of differential splicing and antisense transcripts were confirmed for specific transcripts and the regulatory role of one antisense transcript for the transcription factor *meaB*, investigated. These data have assisted in genome annotation, including the identification of novel genes: we estimate that the total gene number for *A. nidulans* is approximately 11,800, over 1000 more than had previously been annotated. These data have been made available to the *Aspergillus* genome database (AspGD) to facilitate on-going gene annotation and can be visualised via their genome browser.

## Results

### Whole transcriptome sequencing of Aspergillus nidulans

To obtain an accurate representation of the *A. nidulans* transcriptome, we performed high-throughput RNA-sequencing of the wild-type strain G00 under a range of growth conditions [6, 28]. Of 304,193,731 reads 62,213,473 (20.5%) mapped to the reference genome, the majority mapping to only one position. 79.4% of the reference genome assembly was covered by at least one sequence read. Mapping statistics are shown in Additional file 1.

The CADRE genome assembly release 5 has 10827 annotated loci with unique identifiers. Of these, 10530 had an annotated coding sequence, indicating the translated region of a protein coding gene. The remainder consisted of putative pseudogenes and non-coding RNA genes. For all 10827 annotated loci, the number of mapped reads from each sequence library which aligned in the sense and antisense orientations were counted. These counts were normalised by library and locus size to give the standardised measure of expression ‘reads per kilobase per million mapped reads’ (RPKM). These data are shown in Additional file 2. Across the five libraries, 94% of annotated loci had at least one read aligned in the sense orientation and 79% had RPKM > 1 (Additional files 1 and 2), indicating that the majority (80–95%) are expressed under the growth conditions analysed.

### Identification and analysis of novel transcripts

The transcriptome alignment was used to define transcribed regions of the genome (Additional file 3). Using the merged data from all five libraries 44,479 transcribed fragments were defined. This is four times greater than the number of annotated loci. A proportion of these may relate to unanotated loci. However, multiple small fragments can be inferred from what is in fact one transcript due to uneven mapping; a particular problem if the transcript is expressed at a low level. Additionally, alternative splicing may produce multiple transcripts from the same locus.

To estimate the number of putative novel transcripts in the genome, the locations of the 44,479 transcribed regions were compared to the annotated loci. 29% (13026/44479) did not overlap an annotated locus in either direction. On average these were shorter than transcribed fragments that overlapped annotated loci (median length 135 bp and 580 bp, respectively; Additional file 1). The proportion of the transcribed genome associated with these novel transcripts was 8.3%. Assuming annotated and unannotated genes have a similar length distribution, these data suggest that the 10,827 annotated loci represent approximately 92% of all loci. The genome should therefore contain approximately 980 additional transcripts not included in the CADRE release 5 annotation.

To further analyse these unannotated loci, we searched for similarities to known functional non-coding RNAs or protein domains. To identify functional non-coding RNAs, the nucleotide sequences of the 13,026 putatively novel transcribed fragments were used to query the Rfam database [29]. To identify unannotated protein coding genes, we used all open reading frames longer than 300 bp (i.e. 100 aa) within these transcripts (1899 putative peptide fragments derived from 1129 different transcribed fragments) to query the Pfam database [30]. 343 transcripts had at least one match to the Pfam or Rfam database, the results are shown in Additional file 4. A few putative functional RNAs were identified, among them several tRNAs, small nucleolar RNAs and spliceosomal RNAs. Many protein domains were also identified. Since this analysis, many of these genes have been added to later releases of the annotation, in some cases guided by these data. Putative transcripts that remain unannotated at the time of writing include those encoding putative methyltransferase and P450 domains, as well as numerous DDE domains often associated with transposons.

### Introns and alternative splicing

The transcriptome alignment identified 22,636 introns supported by one or more sequence reads (the locations of all identified introns from all sequence libraries are in Additional file 5). The CADRE release 5 annotation contained 24,894 annotated introns, of which 15,870 (63.8%) were confirmed by the transcriptome data. 1087 introns matched the annotated intron only at its 5′ junction and 542 only at its 3′ junction, while 714 overlapped an annotated intron without matching either junction. 4092 did not in any way correspond to an annotated intron.

Introns are generally short (median length 59 bp, with 95% of introns between 46 and 228 bp) but some long introns do exist. For example, an annotated intron of approximately 1.4 kb in gene AN4076 was confirmed by RNA-seq and validated by RT-PCR (data not shown). 21,794 introns had bases GT and AG at their 5’ and 3′ ends, 428 had AT and AC and 414 had GC and AG. The GT-AG introns showed standard consensus sequences at their 5′ and 3′ ends: GTRAAGT (base frequencies: 100, 100, 67, 42, 86, 62%) with conserved AG (40, 56%) immediately upstream at the 5′ end and YAG (90, 100, 100%) at the 3′ end. In all, 18,782 introns were fully contained within 7191 annotated genes. 227 introns extended 191 annotated genes at the 5′ end. 147 introns extended 140 annotated genes at the 3′ end. 18 introns apparently completely spanned 12 annotated loci (Additional file 1). Of these 12 loci, six were snoRNAs and two were 5.8S rRNA. The remaining four were putative protein coding genes, but the long introns could not be confirmed by RT-PCR (data not shown). Careful examination of these loci suggests that the introns are likely to represent mapping artefacts due to the respective genes being flanked by similar tRNA genes.

Alternative splicing increases the diversity of the transcriptome and proteome without the need for additional genomic sequence. A small number of examples of alternative splicing have been described in the Aspergilli [31–35]. Whole transcriptome analysis of *A. oryzae* (which was not strand specific and therefore could not account for the presence of antisense transcripts) reported 8.55% of genes displaying differential splicing with the majority of these events (91.56%) being due to intron retention [21].

**Figure 1:**
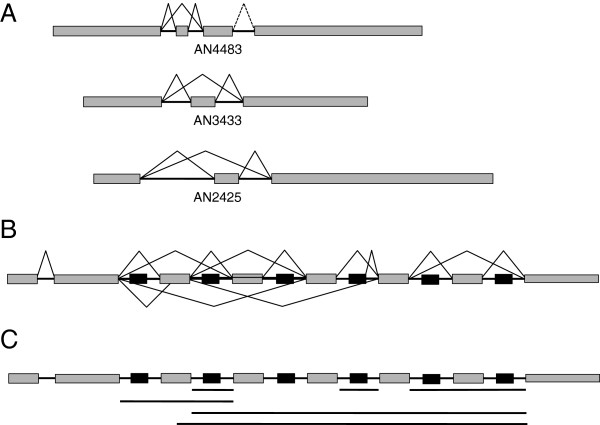
**Alternative splicing of** ***A. nidulans*** **transcripts. (A)** Three examples of exon skipping/retention identified by whole transcriptome mapping and experimentally verified. **(B)** An extreme example of alternative splicing: a putative snoRNA cluster on chromosome V. Black boxes represent putative snoRNAs, grey boxes represent exons. Solid lines represent splicing events verified by RT-PCR. **(C)** Intron retention (solid lines beneath diagram) verified by RT-PCR and sequencing in the snoRNA cluster. The cluster displays a complex combination of splicing, intron retention and exon skipping.

We searched for cases of alternative splicing by comparing transcripts defined by the Cufflinks program. Table 1 summarises the data from Additional file 3. Based on these data, the proportion of alternatively spliced transcripts is low (8.3%) and is broadly similar to that reported for *A. oryzae*[21]. We searched for cases of exon skipping/retention (two non-overlapping short introns each sharing a 5′ or 3′ boundary with a single longer intron) but found only 60 examples of this pattern, indicating that it is rare. Three of these cases were confirmed by RT-PCR (Figure 1A): AN4483, AN3433 and AN2425. We observed that many of the cases of alternative splicing involved intron retention, similar to the case in *A. oryzae*.

**Table 1:** **Alternatively spliced transcripts**

Growth condition	Total transcripts	Transcript sets^a^	Alternatively spliced transcript sets (%)^b^
Nitrate	35167	33934	883 (2.6%)
Complete	34292	33095	868 (2.6%)
Ammonia	36810	35803	715 (2.0%)
-N, 4 h	31847	30552	932 (3.1%)
-N, 72 h	37097	34607	1743 (5.0%)
All	44479	38750	3226 (8.3%)

^a^A set of one or more different transcripts occupying a common genomic locus and sharing regions of a common template sequence.

^**b**^Transcript sets of more than one transcript (as a percentage of all transcript sets).

We observed an extreme example of alternative splicing on chromosome V. This transcript spanned three annotated loci: a putative pseudogene, a putative protein coding gene (AN11485) and a snoRNA. The putative protein coding gene was not supported by the data (no reads aligned to the same strand or supported its introns) but the snoRNA corresponded to a peak of high read coverage. The transcript was unusual in that it contained seven introns each with a peak of high read coverage within it. The alignment indicated alternative splicing with both exon skipping and retention, which were confirmed by RT-PCR. Searches of Rfam indicated that the intra-intron peaks were indeed snoRNAs and the locus appears to be a snoRNA cluster similar to that described in *Neurospora crassa*[36]. Figure 1B illustrates the observed alternative splicing. We could not reconstruct an open reading frame from the exon sequences, suggesting that the function of the transcript is as a precursor to fully processed snoRNAs and the processed mature transcripts may not have an additional functional role.

### Natural antisense transcripts (NATs)

Inspection of the *A. nidulans* transcriptome data revealed a large proportion of genes with strand-specific reads aligned in both the sense and antisense orientations, indicating the existence of many natural antisense transcripts. These represent a subset of non-coding RNAs [37] with potential regulatory roles mediated via various mechanisms including transcriptional interference, chromatin remodelling, RNA interference and translational repression [14]. Examples have previously been identified in *A. flavus* and *A. niger*[16, 38].

Loci with sequence reads mapping in the antisense orientation were relatively common, with 72% having at least 1 mapped read, compared to 94% of loci in the sense orientation (Additional file 1). However, in the antisense orientation only 14% of annotated genes had an RPKM > 1, compared to 74% in the sense orientation. This indicates that antisense transcripts are expressed at a much lower level than its sense counterpart and/or that they cover only a part of a given gene.

To describe the distribution of antisense transcription across genes, we counted antisense reads aligned to the 5′, central and 3′ regions of each annotated gene. Of the 72% of genes (7697/10697) with one or more reads mapped in the antisense orientation under one or more growth conditions (Additional file 6), 352 showed 5′-biased and 352 centrally-biased antisense transcription, but the majority, 1280, showed 3′ bias. This excess of antisense transcription at the 3′ end of genes is consistent with previous findings in *Saceromycese cerevisieae*[39].

Using RT-PCR with oligo dT in combination with gene specific primers we experimentally verified antisense transcripts overlapping four genes: AN8048, AN8040, AN4023 and AN4058. PCR products were cloned and sequenced, confirming the presence of each of the previously unannotated transcripts in the antisense configuration with a known gene.

**Figure 2:**
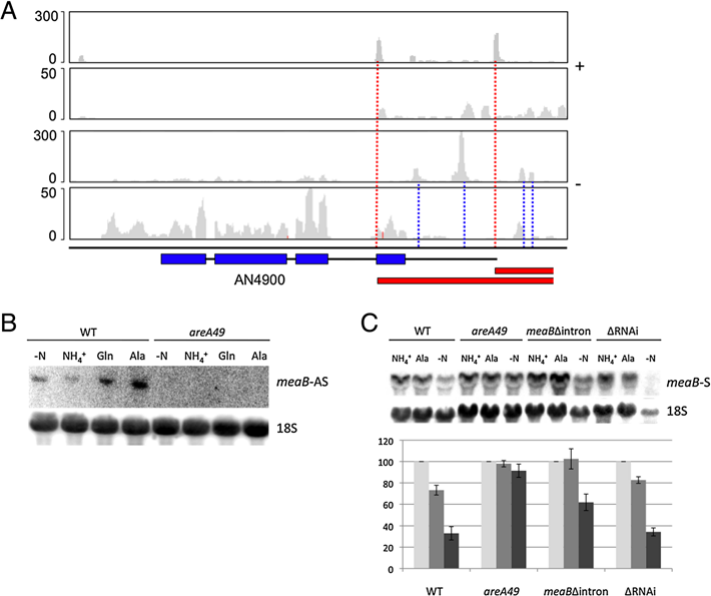
**Antisense transcription at the** ***meaB*** **locus. (A)** Antisense transcription was identified overlapping the 5′ end of the *meaB* gene. Coverage on the positive strand for 5′-specific and whole transcriptome RNA-seq libraries is shown in the top two plots. Coverage on the negative strand for these libraries is shown in the bottom two plots. The *meaB* gene (AN4900) is encoded on the negative strand (blue boxes and thin black lines represent exons and introns/UTR, blue dotted lines indicate multiple transcription start sites). Red boxes indicate predicted antisense transcripts, their transcription start sites shown by red dotted lines. **(B)** From northern analysis of the wild type (WT) the antisense transcript (*meaB*-AS) was identified and found to be modulated by nitrogen regime; NH_4_^+^, glutamine (Gln), alanine (Ala) as sole nitrogen source or nitrogen starvation (-N), for two hours. Analysis of a strain bearing a loss of function mutation in *areA* (*areA49*) revealed that *meaB*-AS expression is AreA dependent. 18S rRNA was used as a control. **(C)** Quantitative northern analysis of the *meaB* sense transcript (*meaB*-S) in the wild type (WT), *areA49*, *meaB*Δ*intron* and Δ*RNAi* strains was undertaken. The mean intensity, relative to WT, of four independent experiments is plotted (+/- SD). Downregulation of *meaB* transcript levels in response to poor nitrogen (Ala) was lost in the *meaB*Δ*intron* strain but the response to nitrogen starvation was retained.

We investigated one example of antisense RNA in more detail. The transcription factor *meaB* (AN4900) has a regulatory role associated with nitrogen availability [40, 41]. Examination of RNA-seq data revealed an antisense transcript which initiates at a position aligned to the first intron of the sense transcript. The intron sequence, which is relatively long at 331 bp compared to a median intron length of 59 bp, contains six GATA motifs, indicating a possible functional association with a second transcription factor, AreA [42], which has been shown to regulate *meaB* transcript levels [41]. To confirm the presence of the antisense transcript and test the possibility that it is under the regulation of AreA, we utilised northern analysis using a single stranded probe from exon 1. As shown in Figure 2, the antisense transcript is differentially regulated in response to nitrogen regime and its expression is dependent on functional AreA. To investigate the function of the *meaB* antisense transcript we specifically deleted the first intron of *meaB* by homolgous integration at the *meaB* locus. Northern analysis revealed that modulation of *meaB* transcript levels was largely lost, except under nitrogen starvation (Figure 2). We also found that a strain disrupted for three loci potentially involved in RNAi [43] did not show significant altered regulation of *meaB* transcript levels, indicating that RNAi is not responsible for antisense mediated regulation of *meaB*.

### Identification of transcription start sites and analysis of 5′ untranslated regions and promoters

In order to further characterise the *A. nidulans* transcriptome and help improve gene annotation, we constructed RNA-seq libraries enriched for 5′ TSS, utilising an approach based on 5′ RATE [26]. The mRNA was decapped using tobacco acid pyrophosphatase (TAP) prior to ligation of adapters to the unfragmented RNA and reverse transcription, using a primer containing a random hexonuleotide sequence at its 3′ end. The products were size selected, amplified by PCR and sequenced using the conventional SOLiD protocol. The resulting 5′ nucleotide position of each read, here referred to as the ‘read head’ (RH), was mapped to the genome assembly and the number of read heads at each position counted, peaks representing putative 5′ ends of transcripts.

**Figure 3:**
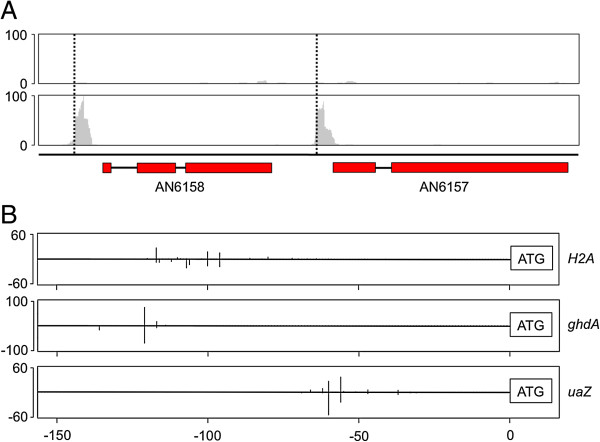
**5′-specific RNA-seq locates transcription start sites. (A)** Coverage depth of 5′-specific RNA-seq libraries (plotted in grey). Peaks and dotted lines indicate the transcription start sites of two annotated genes: AN6158 and AN6157. **(B)** Experimental validation of 5′-specific RNA-seq data. The x-axis indicates the distance (in base pairs) upstream of the translation initiation codon ATG for three genes: *H2A.Z* (AN8039), *gdhA* (AN4376) and *uaZ* (AN9470). For each gene, the proportion of RNA-seq reads starting at a given position is plotted above the line and the approximate proportion of 30–50 Sanger-sequenced plasmids containing products of circularisation RT-PCR supporting a given TSS is potted below the line.

The initial 5′ sequencing library (‘TAP-1’) consisted of 64,148,556 reads, of which 18,817,969 (29.33%) mapped uniquely to the reference genome (Additional file 1). Inspection of the sequence alignment showed a high frequency of RH mapping close to the 5′ ends of annotated genes, indicating successful 5′ enrichment. Additionally, widespread low level mapping was observed, possibly due to the sequencing of fragmented and partially degraded transcripts. To assess this we made a library in which the mRNA was pre-treated with alkaline phosphatase (AP) prior to decapping, in order to prevent fragmented or degraded RNA from ligating to the 5′ PCR primer. Additionally we made a library without pre-treatment of the RNA with TAP, thus eliminating capped mRNA from the sequence library. In the untreated library, low levels of reads were distributed throughout the transcribed regions with no peaks generally observed at the 5′ ends of annotated genes. Some peaks of high coverage were evident in the untreated library. These were often associated with the 5′ ends of putative snoRNAs (Additional file 7). In contrast, TAP-treated libraries showed discontinuous read distributions with peaks of coverage commonly occurring upstream of annotated genes (Figure 3A). The accuracy of the transcript 5′ end mapping was assessed by comparing it with circularisation RT-PCR or 5′ RACE data, which specifically defines the 5′ ends of transcripts. This analysis showed agreement between the methods, as illustrated in Figure 3B.

For further analysis, to maximise the depth of coverage and identify as many putative TSS as possible, the four TAP-treated libraries (TAP-1, TAP-2, TAP-3 and AP + TAP) were combined. Using a region of 121 bp containing a minimum of 50 RH and a minimum major peak of 10 RH, we defined 17992 putative transcription start site regions. We developed a metric to measure the distribution of reads across each TSS region, based on a confidence interval around the major TSS (described in the Methods section). This was used to define three sets of TSS regions. The first contained 4557 putative transcription start sites with a very tight distribution or strong single start site where the 95% confidence interval was within two nucleotides. The second contained 4637 less specific single start sites (95% confidence intervals >2 and <4). The third contained the 8798 most diffuse TSS (95% confidence intervals >4). Of the tight, intermediate and diffuse TSS regions, 72.3% (3293), 46.8% (2171) and 25.0% (2197) were within 500 bp upstream of annotated genes. Conversely, 11.9% (542), 23.4% (1087) and 40.9%% (3595) occurred fully within annotated genes, suggested that the tight TSS regions are likely to be enriched for real TSS. However, the diffuse TSS regions might include a proportion of artefacts arising from degradation of mRNA. All putative promoters are recorded in Additional file 8 and all identified TSS located within 500 bp of the 5′ end of an annotated gene and in the correct orientation are listed in Additional file 9. According to our classification of TSS, 3293 ‘tight’ , 2172 ‘intermediate’ and 2197 ‘diffuse’ putative TSS were associated with annotated genes.

**Figure 4:**
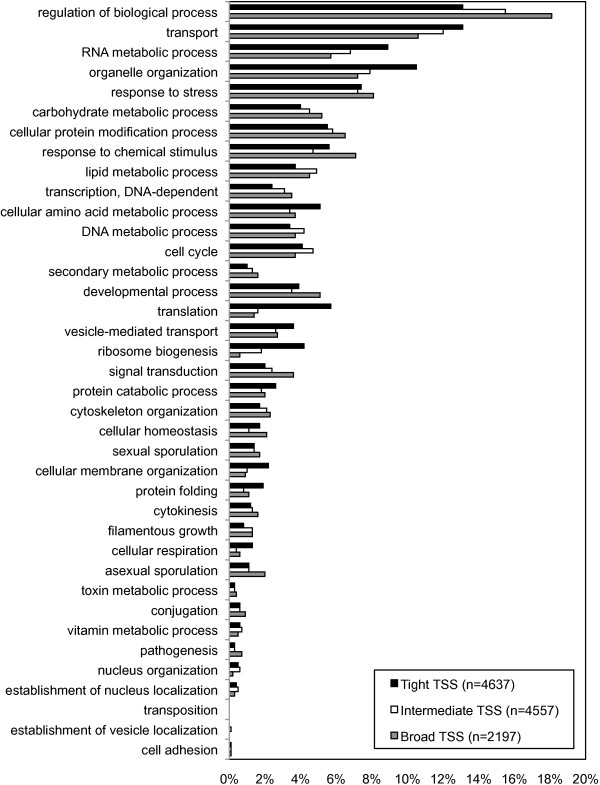
**Functional description of genes associated with different TSS classes.** The proportions of gene sets associated with different classes of TSS (‘tight’, ‘intermediate’ and ‘diffuse’) with general functional gene ontology descriptions. In total, 4637, 4557 and 2197 genes were asociated with ‘tight’, ‘intermediate’ and ‘diffuse’ TSS, respectively. Of these genes, the proportion of each set associated with a given GO term is plotted (note that genes can be associated with more than one GO term). Differences among the frequencies of GO terms reflect their overall frequency distribution across the genome. In addition, the frequencies of some functional descriptions vary among the different TSS classes. For instance, translation and ribosome biogenesis are distinctly skewed towards genes with tight TSS.

The association of specific gene functions with different classes of promoter was investigated. Figure 4 shows the proportion of each of the three gene sets assigned a broad functional description. Genes associated with ‘tight’ TSS are enriched for functions related to translation and ribosome biogenesis, with genes associated with diffuse TSS tend to be enriched for regulatory processes and signal transduction. The full table of GO terms significantly enriched for each gene set is in Additional file 10.

**Figure 5:**
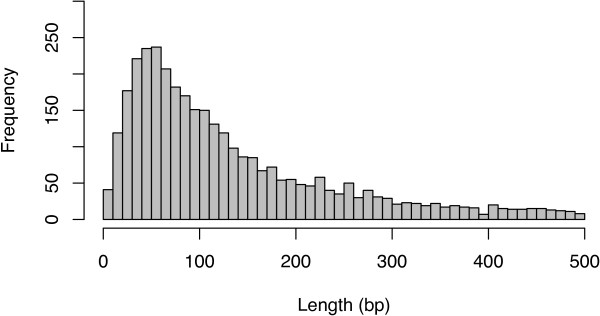
**5′ UTR lengths.** Length distribution of 5′ untranslated regions (between the transcription start site defined by 5′-specific RNA-seq and the annotated translation initiation codon) for ‘tight’ transcription start sites.

**Figure 6:**
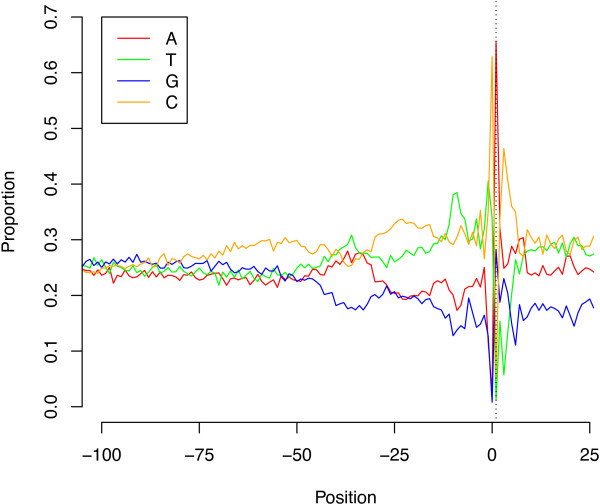
**Base composition around transcription start sites.** The proportions of bases from 100 bp upstream to 25 bp downstream of putative 4637 ‘tight’ transcription start sites (100-200 bp upstream shows no perturbation from the 25% expected by chance and is not shown). The region is enriched for C from approximately position -75 and for C and T from approximately position -50. A peak of A and T is seen in the region -45 to -30, which may indicate putative TATA box motifs. Nucleotide C peaks at position -1 and nucleotide A at the transcription start site, followed by C.

**Figure 7:**
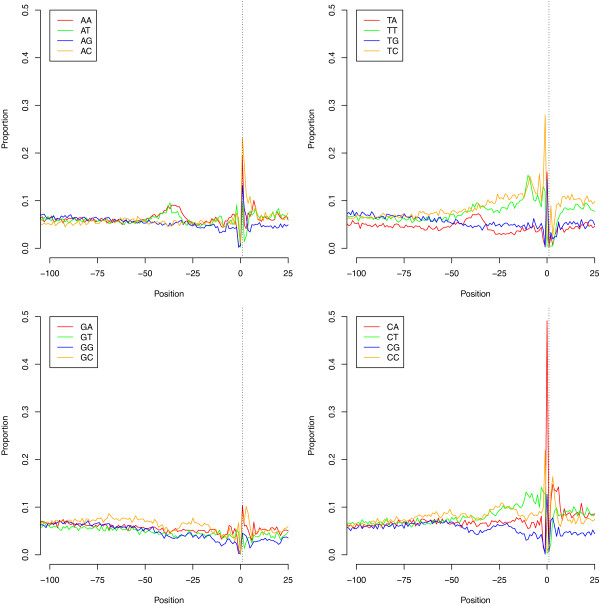
**Dinucleotide composition around transcription start sites.** The proportions of all possible dinucleotides from 100 bp upstream to 25 bp downstream of 4637 putative ‘tight’ transcription start sites (100-200 bp upstream shows no perturbation from the 6.5% expected by chance and is not shown). A small peak in AA, AT and TA frequencies in the region -45 to -30 may represent the TATA box. From -50 to +25 around the transcription start site is enriched for pyrimidine pairs (TT, TC, CT, CC) and the TSS and position -1 is strongly enriched for CA, reflecting the single nucleotide frequencies at these positions.

To define the length of 5′ UTRs, the distance from each defined transcription start site to the nearest downstream annotated translation start codon (up to a maximum of 500 bp away) was plotted (Figure 5). The majority of ‘tight’ TSS were followed by a 5′ UTR of less than 100 bp (median 93 bp, with 95% of UTR between 15 and 434 bp). However, a proportion of genes with long 5′ UTR would not be detected here, as we set a maximum distance of 500 bp from TSS to translation start codon to minimise false positive results. For the more diffuse TSS, the distribution 5′ UTR length was much broader (data not shown). To characterise upstream promoter regions, we analysed regions 200 bp upstream and 25 bp downstream of the putative TSS. Figure 6 shows the base composition and Figure 7 the dinucleotide composition of these regions for the 4637 ‘tight’ TSS (data not shown for the less well defined ‘intermediate’ and ‘diffuse’ TSS sets, but are broadly similar to data for the ‘tight’ TSS set). The plots indicates that base and dinucleotide composition is skewed within approximately 75 bp immediately upstream of the TSS, indicating that functional elements of the core promoter may lie within this region. Further upstream (position -100 to -200) showed no major skewed base/dinucleotide frequencies (data not shown). The major features that can be discerned include a C-rich region extending approximately from position -75 to -50 and a generally pyrimidine (TC)-rich region spanning the TSS from -50 to at least position +25. Immediately upstream of the TSS, C is the most common nucleotide (62.9% of tight promoters), followed by T, A and G (33.9%, 2.4% and 0.8%). At the TSS itself, A is the most common base (65.7% of tight promoters), followed by G, C and T (28.1%, 5.0% and 1.2%). Reflecting this, CA is the most frequent dinucleotide spanning the TSS (49.1% of tight promoters), followed by TA, TG and CG (16.0%, 14.4% and 12.7%). All other dinucleotides here are each at less than 5% frequency. Other peaks include TT/TC approximately -10 bp upstream of the TSS and an A and T rich peak in the region approximately -45 to -30 bp upstream of the TSS, which may represent the site of the TATA box.

**Figure 8:**
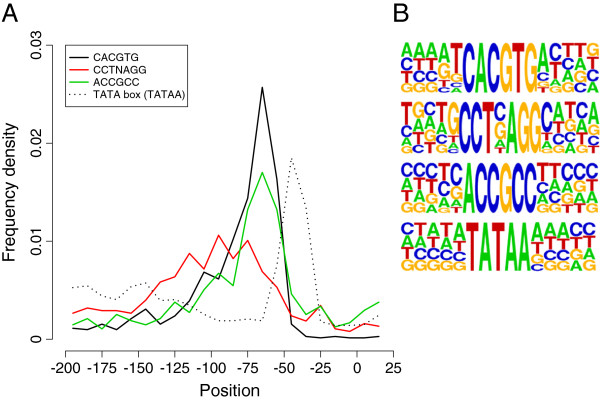
**Distribution of putative promoter motifs relative to transcription start sites. (A)** Distributions of consensus motifs CACGTG, CCTNAGG and ACCGCC within promoter regions of ‘tight’ transcription start sites. The distribution of putative TATA box motifs (TATAA) are also shown. Each point represents the frequency density of motifs starting within a 10 bp window, the midpoint of which is plotted. Both CACGTG and ACCGCC peak at approximately -70 bp and the TATA box at -45 bp from the TSS, while the position of the CCTNAGG motif is less sharply defined across a region from approximately -70 bp to -120 bp. **(B)** Sequence logos of promoter motifs and surrounding nucleotides (from promoter regions associated with ‘tight’ TSS).

To identify functional motifs in the upstream promoter regions, we searched for statistically over-represented strings of bases within the promoters of tight, intermediate and diffuse TSS using the program YMF [44]. Similar results were obtained from the program MEME (data not shown) [45, 46]. The full results from YMF are shown in Additional file 11. From this analysis, we identified a large number of variants on three common sequences: CACGTG, ACCGCC and CCTNAGG. We investigated the distribution of these motifs relative to the 4637 ‘tight’ TSS (Figure 8) and each was clearly not uniform (chi-squared test of uniformity: P < 2.2x10^-16^ in all cases) occurring primarily more than 50 bp upstream of the TSS. The marked non-random distribution of these putative motifs relative to the TSS suggests that their location is relevant to their function. Our analysis of enriched motifs did not identify known core promoter motifs such as the TATA box (TATAA) so we searched for this motif individually in the promoter regions (Figure 8). The distribution of putative TATA box motifs was strongly positionally specific, with the majority occurring 30–45 bp upstream of the TSS, consistent with the skewed A and T frequencies in this region. We also searched for motifs including the CAT box (CCAAT), initiator element (YYANWYY), heat shock elements (NGAANNTTCN) [47] and regulatory motifs bound by CreA (SYGGGG) [48] and AreA (HGATAR) [49]. Their positions were generally much less well defined than for the other motifs although the InR and CAT box tended to be more common near to the TSS while the CreA and AreA motifs tended to be more common between -50 and -100 (Additional file 1).

**Figure 9:**
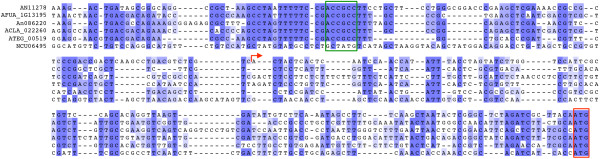
**Conservation of motif ACCGCC among** ***Aspergillus*** **species.** Sequence alignment of the region upstream of the putative translation start codon ATG (boxed in red) of genes orthologous to *A. nidulans* AN11278 in five species of *Aspergillus* (*A. nidulans*, *A. fumigatus*, *A. niger*, *A. clavatus*, *A. terreus*) and *Neurospora crassa*. The major *A. nidulans* transcription start site is indicated by a red arrow. The ACCGCC motif (boxed in green) is conserved among Aspergilli, though not in *N. crassa*.

To further validate the putative motifs CACGTG, CCTNAGG and ACCGCC, we investigated whether they were conserved among the distantly related species *Aspergillus nidulans* and *Aspergillus fumigatus*. We defined 5872 orthologue pairs between the two species and utilising chi-squared analysis determined if co-occurrence in orthologous promoters was significant. For all three motifs this was the case (CACGTG, p < 2.2 × 10^-16^; ACCGCC, p < 2.2 x 10^-16^; CCTNAGG, p = 3.17 × 10^-10^). This is consistent with all three motifs being functionally conserved. Sequence alignment of upstream regions for orthologous genes from multiple species also illustrates the phylogenetic conservation of motifs (Figure 9 and Additional file 1).

## Discussion

We carried out sequencing of both the whole transcriptome and transcript 5′ ends from the filamentous fungus *A. nidulans* using five different growth conditions. Combining these two methods provides a useful way to define transcript structure and to improve genome annotation.

We observed that at least 80% of the reference genome assembly was transcribed across all five conditions tested. While these proportions should be treated with some caution as they are sensitive to factors such incomplete sampling by smaller sequence libraries, such effects will lead to underestimation so we may still conclude that the majority of the genome is transcribed. Furthermore, this will inevitably be extended when further growth regimes are examined. The developmental state of the cultures was not measured but some growth conditions, particularly nitrogen starvation, can induce sporulation in submerged cultures [50]. Our data suggest that this is the case, as *brlA* and several other genes up-regulated during condia formation [51] appear to be upregulated in cells under long term nitrogen starvation (Additional file 2). This may in part explain why more of the genome (approximately 71%) appears to be transcribed under nitrogen starvation compared to other conditions (54-61%). However, for all conditions, more than 50% of the genome was transcribed. The annotated protein coding portion of the genome is approximately 50%, indicating a large amount of functional non-coding RNA in addition to this (including functional non-coding transcripts in addition to untranslated regions of protein-coding genes). In this, *A. nidulans* resembles *S. cerevisiae*, in which approximately 75% of the genome encodes proteins but 85% of the genome is transcribed [39]. More extreme pervasive transcription is displayed by mammalian genomes (e.g. [52–55]), in which the difference between protein coding sequence (generally <5% of the genome) and total transcribed sequence (70-90%) far exceeds that seen in the more gene-dense fungi.

**Figure 10:**
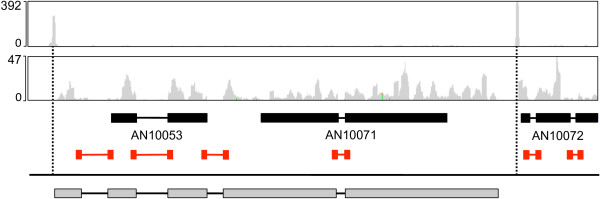
**Gene annotation improvement guided by RNA-seq.** Example of a correction of misannotated gene models. In the CADRE release 5 annotation two genes, AN10053 and AN10071, were predicted (thick and thin black bars represent exons and introns). Whole transcriptome (middle track, coverage depth plotted in grey) and 5′-specific RNA-seq (top track, coverage depth plotted in grey) revealed the general structure of transcripts produced in this region. Whole transcriptome coverage identified four introns in the region covering the two genes (red), two previously unidentified introns extended AN10053. 5′-specific sequencing identified a single 5′ end for both annotated genes (left dotted line). The combined data indicates a single gene (bottom, grey boxes and black lines represent exons and introns).

Updating and improvement of the *A. nidulans* genome annotation is an ongoing process and combining whole transcriptome sequencing with 5′ specific RNA-seq data can help to improve gene annotation. As an example, a gene which had been mis-annotated as two separate loci was identified due to the occurrence of only a single TSS and identification of previously unannotated introns (Figure 10). To aid the annotation process, the RNA-seq data were made available to the research community via the CADRE and AspGD websites [7, 8]. At AspGD, read alignments can be viewed (using the JBrowse feature on each gene page) and the data have been used to update many of the gene model annotations on AspGD (where this is the case is indicated in the ‘locus history’ section of the gene page). Consequently, the current AspGD annotation has changed considerably since that analysed here. For instance, we found many putative unannotated protein genes in our analysis and estimated that the total number of transcribed genes is close to 11,800 (approximately 1000 more genes than in the annotation we analysed). Many of these putative genes have since been annotated.

Transcriptome complexity was extended by many examples of alternative splicing. Predominantly this involved intron retention which we found generally resulted to the inclusion of an in-frame stop codon into the transcript. Therefore, we hypothesise that intron retention is not primarily a means of generating novel proteins, but is linked to repression of translation. This could be achieved by rapid nonsense mediated decay of transcripts containing premature stop codons [56], or intron retention could prevent export of the transcript from the nucleus [57].

We observed many instances of natural antisense transcripts (NATs). NATs are potentially functional long non-coding RNAs that may regulate protein expression via a number of possible mechanisms: transcriptional interference, chromatin remodelling, and double-stranded RNA formation leading to translational repression, RNAi or disrupting mRNA maturation and processing. A number of transcriptome analyses have identified NATs in fungi [16, 18, 58, 59]. Up to 90% of *S. cerevisiae* genes show some level of antisense transcription and the pattern of antisense transcription appears to be evolutionarily conserved among multiple yeast species, suggesting an important conserved gene regulatory mechanism [58, 59]. In *A. nidulans* we found that 72% of genes have at least some antisense transcription, with 14% showing this to be relatively abundant (>1 RPKM). Despite the importance of antisense transcripts suggested by this ubiquity and conservation, validated examples of antisense mediated regulation are limited, although artificial antisense constructs have been used to knock down gene expression in various species including *A. nidulans*[60]. In this respect we have presented data for the *meaB* loci, which supports the model whereby the antisense transcript is upregulated when the sense transcript is repressed and that elimination of the antisense transcript leads to loss of this transcriptional regulation. With regards to the regulatory mechanism involved in this case we found that disruption of the genes associated with RNAi [43] did not have an impact on *meaB* transcript levels. From our data in relation to *meaB* there is no evidence for splicing being affected. It is interesting to note that the orthologous gene in *Fusarium fujikuroi*, has a similar, relatively large intron containing GATA motifs and a functional promoter [41]. In this case the resulting short transcript, which is not likely to code a functional protein, is also upregulated under nitrogen limitation but is transcribed in the sense and not the antisense direction. The apparent functional conservation would possibly support chromatin structure playing a key role in this regulatory mechanism.

The prevalence of antisense transcripts has important consequences when monitoring gene transcription. Conventional northern or quantitative PCR (qPCR) approaches do not take this into account, although in the case of northerns it is likely that the different forms of transcript identified by a probe will be distinguished on the basis of size. However, qPCR will generally not distinguish between sense and antisense unless the primers are specifically designed to avoid antisense transcripts on the basis of location or splicing patterns. As the sense transcript and antisense can be differentially regulated, very significant errors in calculating transcript levels may result as a consequence.

5′-specific RNA-seq allowed us to identify a large number of putative transcription start sites. A range of patterns of TSS distribution was seen, from tightly defined TSS with a single, well supported position to more diffuse collections of individually less well supported TSS. The occurrence of multiple distinct TSS, and consequent 5′ heterogeneity, has been described in many eukaryotic organisms, including fungi [61–65]. We divided putative TSS into three groups: ‘tight’ , ‘intermediate’ and ‘diffuse’, based on the distribution of reads. RNA polymerase II-dependant promoters of vertebrates have previously been divided into two groups, categorised as either TATA or CpG types, the former with a tight TSS a strict distance from the TATA box and the latter with broad clusters of TSS [62, 66]. The *Drosophila melanogaster* genome has both tight and broad TSS clusters, tight promoters appear to be enriched for core promoter elements such as the TATA-box, however *Drosophila* lacks CpG islands, therefore its broad promoters are not CpG-associated [67]. In *Arabidopsis thaliana* the TATA promoter is also associated with sharp TSS clusters [68]. In our analysis of *A. nidulans*, we found putative TATA-box motifs (TATAA) upstream of 21% of ‘tight’ TSS, 17% of ‘intermediate’ TSS and 11% of ‘diffuse’ TSS, approximately 30 to 45 nucleotides upstream of the start site, suggesting some enrichment in tight promoters.

To analyse the core promoter regions, we focused our study on 4637 well defined ‘tight’ TSS. The core promoter serves as the site of organization of the basal transcription machinery, including RNA polymerase II. Based on the defined transcription start sites, we analysed upstream regions to characterise these putative promoters. Based on the skewed distribution of nucleotides, we suggest that the promoter regions are quite restricted, commonly extending no further than 75 bp upstream of the transcription start site. This is consistent with the relatively small intergenic regions of *A. nidulans*, compared to, for instance, mammalian genomes. These core promoters are generally enriched for pyrimidines (particularly between position -50 and -1 and also in the 5′ UTR to at least 25 bp downstream of the TSS) and an A and T rich region between -45 and -30 may represent the TATA box. The pyrimdine rich sense strand upstream of the TSS has been noted previously [69]. Most strikingly the TSS are commonly defined by the dinucleotide CA (49% of ‘tight’ TSS), with A as the first base of the transcript. There are no universal core promoter motifs identified in eukaryotes, but some, such as the TATA box, are present in 30-40% of promoters in higher eukaryotes. The TATA box and initiator (Inr) element tend to have a relatively fixed location upstream of genes [70]. Although neither of these elements were identified by *de novo* analysis of promoter sequences based on motif enrichment they did show distinct distributions which are indicative of their functional role within promoters and also supports validity of our data.

We identified a small number of putative core motifs that were over-represented in promoters and showed a strong, significant bias in their location upstream of the TSS. They also appeared to show greater than expected conservation among divergent *Aspergillus* species (e.g. *A. fumigatus* and *A. oryzae* proteins share approximately 66-67% sequence similarity with *A. nidulans*[6]). Taken together, this is consistent with them being promoter elements. Further experimental analysis will be required to assess their functions – key to this will be whether they are involved in modulation of promoter activity or are, like the TATA box, primarily associated with the core function, defining the promoter start site.

## Conclusions

Transcriptome sequencing, including 5′ specific analysis, has revealed the complexity of the *A. nidulans* transcriptome. We found alternatively spliced genes, intron retention and many cases of overlapping transcripts. In one example, *meaB*, we have shown the antisense RNA has a regulatory role. We also identified many previously unannotated genes. Global analysis of 5′ transcript ends provides an extensive map of promoters across the genome, defining key features and facilitating future analysis of promoter structure and function.

## Methods

### Growth of Aspergillus nidulans and RNA extraction

All analyses used the *Aspergillus nidulans* wild-type strain designated G00 in the Glasgow collection. This is the strain used for the genome sequencing project (in that case named FGSC A4) [6]. Mycelia were grown in an orbital incubator at 190 rpm for 16 hours at 37°C in batch cultures of 250 ml under five different growth conditions: complex media or synthetic minimal media with nitrate as the sole nitrogen source; ammonia as the sole nitrogen source; nitrogen starvation for four hours; nitrogen starvation for 72 hours. Both minimal synthetic or complete media [71] were used with glucose (1% w/v) as sole carbon source. For minimal media nitrogen was supplied as either NaNO_3_ (nitrate) or ammonium tartrate (10 mM). For nitrogen starved cultures initial growth was conducted over night with nitrate. The mycelia were then harvested by filtration through Miracloth (Calbiochem) and washed with fresh, warm, nitrogen free media prior to re-inoculation and further incubation for either 4 or 72 hours.

Harvested mycelia were washed with cold water, excess liquid was removed on blotting paper and mycelia were snap-frozen in liquid nitrogen. Total RNA was extracted by cryogenic grinding and successive rounds of purification by phenol:chloroform and lithium chloride precipitation as described previously [72]. Poly(A) + RNA was isolated by oligo(dT) selection using the Oligotex direct mRNA kit (QIAgen), following the manufacturer’s protocol.

### SOLiD RNA-seq library preparation

For total mRNA-sequencing libraries, poly(A) + selected RNA was decapped with Tobacco Acid Pyrophosphatase (Epicentre) and fragmented with RNaseIII (ABI). Strand specific cDNA libraries were constructed using oligo-dT selected mRNA and sequencing libraries prepared according to the standard protocol for SOLiD Total RNA-seq and barcoding kits (ABI).

5′ specific RNA-seq libraries were prepared from samples of oligo-dT selected RNA. RNAs were decapped with Tobacco Acid Pyrophosphatase (Epicentre). The RNA was not fragmented prior to SOLiD adaptor ligation which was performed as for Total RNA-seq libraries. By excluding the fragmentation step, full length transcripts containing the SOLiD P1 PCR primer at the 5′ end. cDNA were produced by RT PCR with Superscript III (Invitrogen) and a random primer consisting of the internal adaptor sequence of the SOLiD P2 adaptor with a 5′ hexomeric degenerate sequence (5′-CTGCCCCGGGTTCCTCATTCTCTNNNNNC-3′). The resulting cDNA libraries consisted entirely of random length fragments representing the 5′ end of an mRNA molecule with the full SOLiD P1 adaptor and the internal adaptor sequence of the SOLiD P2 adaptor. cDNA libraries were purified, size-selected and amplified as for Total RNA-seq library preparation and subjected to the maximum recommended number of 18 PCR cycles using reagents from the ABI Total RNA-seq and barcoding kits (ABI).

Additional libraries were prepared to sequence only the 5′ ends of full-length, capped RNAs. The protocol used was as above, with the addition of rAPid alkaline phosphatase (Roche) treatment prior to decapping. Alkaline phosphatase removes the phosphate group from the 5′ ends of RNAs where no cap structure is present, thus excluding natively decapped or fragmented RNAs from the final sequencing library. Reactions were stopped by phenol:chloroform extraction.

### SOLiD sequencing and data mapping

All libraries were aligned to the *Aspergillus nidulans* FGSC A4 genome sequence [6] release 5 (20-05-2010) downloaded from the Ensembl Genomes ftp site [9]. The 5′ TSS-enriched RNA-seq libraries were sequenced on the ABI SOLiD version 3 plus. 5′ TSS sequence reads were aligned to the reference sequence using Bowtie version 0.12.7 [73]. Whole transcriptome RNA-seq libraries were sequenced on the ABI SOLiD version 4. Whole transcriptome sequence reads were aligned to the reference sequence using Tophat version 1.3.1 [74] to allow mapping of reads across splice junctions. Tophat was run initially without any guidance from reference annotation, using default parameters except for a maximum intron length (option ‘-I’) of 4000 and minimum intron length (option ‘-i’) of 10, to disallow the prediction of unrealistically long introns. Predicted introns from the unguided mapping were combined with predicted introns from the annotation and Tophat was run again with these intron locations as a guide. This method ensures that these putative splice junctions are specifically tested and improves intron finding performance.

All sequence read and quality data were submitted to the European Nucleotide Archive under the project accession number PRJEB4484. In addition, the data were made publicly available online through the Central Aspergillus Data REpository (CADRE) website [8, 75] and the AspGD website [7, 76].

### Bioinformatic analysis of novel transcripts and antisense transcription

Transcript prediction based on the Tophat alignment of whole transcriptome data was performed using Cufflinks version 1.3.0 [77]. Alignment files were split to separate reads mapped to the forward and reverse strands and Cufflinks was run, using default parameters, on each file separately. Comparison of predicted transcripts to the *A. nidulans* CADRE release 5 genome annotation (to identify unannotated transcripts) was done using custom perl scripts.

To assess the protein coding potential of unannotated transcripts, open reading frames were identified and translated using the ‘getorf’ program, part of the EMBOSS suite of sequence analysis software [78]. A minimum ORF length of 300 nucleotides in either direction was specified. Translated ORFs were used to search the Pfam database of protein domain hidden Markov models via the web server [30, 79].

To analyse antisense transcription, we measured the distribution of antisense reads aligned to annotated loci. We split each locus into 5′, central and 3′ thirds and defined loci as 5′-biased (>40% of antisense reads mapped 5′, <10% mapped 3′), centrally biased (>75% of antisense reads mapped centrally) or 3′-biased (>40% of antisense reads mapped 3′, <10% mapped 5′).

### Confirmation of alternative splicing

Example of genes for which the RNA-seq alignment indicated multiple transcript isoforms due to alternative splicing were validated by RT-PCR. We designed primers to detect transcript fragments of different length across the putative alteratively spliced region of three genes: AN4483 (forward 5′-CAAGGATGCTTCCGGTGA-3′ and reverse 5′-CAGAGCTCGAGGACAATG-3′); AN3433 (forward 5′-CCAATCAACACCGTCCTC-3′ and reverse 5′-TGCAGGCCATTGACTAGC-3′); AN2425 (forward 5′-TCTGGGGTCTGATGTTCC-3′ and reverse 5′-CAGCGGCTGACGACAAAA-3′). Total RNA was extracted from wild type G00 *A. nidulans* grown on both minimal media in the presence of sodium nitrate as a sole nitrogen source and on nutrient limited media (oat) as an extreme alternative condition where metabolic stress should promote alternative splicing of transcripts. RNA samples were treated with DNaseI followed by phenol/chloroform purification to remove genomic DNA contamination. Reverse transcription was performed using an oligo-dT primer (25mer) and Superscript III reverse transcriptase (Invitrogen), according to the manufacturer’s protocol, followed by PCR. PCR products were run on a 2% agarose gel stained with ethidium bromide and visualised under UV and their sizes determined using a 50 bp ladder.

### Confirmation of novel natural antisense transcripts

RNA extraction and RT-PCR were carried out as described above. Specific primers were designed for transcripts antisense to four annotated genes: AN8048 (5′- GTCCGCCAGATATACTA-3′); AN8040 (5′-GAACTGGCTCTTAATG-3′); AN4023 (5′-ATCACCGAACTGAGACT-3′); AN4058 (5′-CCACCGTATATCATCAG-3′). These were used in combination with an oligo-dT (25mer) primer to amplify approximately 200 bp at the 3′ end of each transcripts. 30 rounds of PCR were performed with and KOD hot-start polymerase (Novagen) according to the manufacturer’s protocols. PCR products were separated on a 1.5% agarose gel and bands around 200 bp length excised and purified using the QIAquick gel extraction kit (QIAgen). DNA fragments were ligated into pGEM-T Easy vectors (Promega) and transformed into competent *E. coli* which were grown on selective media containing ampicillin. Plasmids were extracted using a plasmid extraction kit (QIAgen). Sanger sequencing was used to confirm the identity of the amplified fragments.

### Defining 5′ transcription start sites

Mapped 5′ TSS read coverage data were converted to read head (RH) frequencies. Read heads are the base 1 position up or downstream of the 5′ start position of each read mapped to the genome (the 1 bp offset accounts for the single base trimmed off the 5′ end of the read during mapping). Visual inspection of the alignment indicated that in the majority of cases, a major peak was surrounded by lower peaks within approximately 60 bp on either side. We therefore defined a transcription start site region as a region of 121 bp centred on a major RH peak (minimum depth 10 RH) with a minimum of 50 RH mapped within it. This captured ‘tight’ TSS regions with a single peak, as well as more ‘diffuse’ TSS regions with multiple, lower peaks.

We sought a way to quantify how tight or diffuse the ‘distribution of each transcription start site: whether there was a single, well defined, start site or a series of alternative start sites spread over a given area. If RH frequency peaks were normally distributed around the TSS then the mean would be the main TSS and a confidence interval would define the distribution around it. However, RH frequencies within 121 bp TSS regions were not normally distributed but often a number of randomly dispersed and sized peaks occurred within this window. We found that we could calculate an analogous confidence interval metric if the data were adapted by reversing the region around the peak position and adding the RH frequencies of the reversed region to those of the original to create an approximately normal distribution while maintaining the variance of the unedited data. The relationship between variables could then be expressed by the following equation:

where z is the upper percentage point of the standard normal distribution and confidence interval length (CIL) is directly proportional to the standard deviation of RH distribution (σ) and inversely proportional to the sample size (n). The distributions of adapted TSS regions are still non-normal and many of sample sizes were small, so a bootstrap method, which does not assume any underlying distribution, was applied to evaluate CIL. We used this metric to group TSS regions into ‘tight’ (CIL < = 2), ‘intermediate’ (2 < CIL < = 4) and ‘diffuse’ (CIL > 4).

### Identification of putative transcriptional regulatory motifs

In order to identify putative transcriptional regulatory motifs in upstream promoter regions, we used the TSS data to define promoters. For ‘tight’ (CIL < 2), ‘intermediate’ (2 < CIL < 4) and ‘diffuse’ (CIL > 4) TSS, a region 200 bp upstream and 24 bp downstream of the main TSS (the central, highest RH frequency peak) was analysed. For the diffuse promoters, to further reduce artefacts, only promoters within 500 bp upstream of an annotated gene were analysed.

Putative functional motifs in these promoters were identified using YMF v3.0 [44]. YMF constructs a third-order Markov model to identify motifs enriched in a set of sequences. Enrichment is assessed relative to a ‘background’ based on similar genomic sequence. This background was a set of 10529 sequences of 300 bp upstream of every annotated translation start codon in the genome (translation start codons were used rather than putative transcription start sites as 5′ UTRs are not well annotated in the genome). YMF motif finding was performed for motifs of length 6, 7, 8, 9 and 10 bp, allowing up to 2 ambiguous bases per motif. Each run produced 1000 predicted motifs ordered by Z-score, indicating overrepresentation of the motif against the background. The output of YMF includes many overlapping and therefore redundant motifs. To reduce this redundancy, the Find Explanators program was applied [80], which output <20 motifs for each length (6-10 bp). These motifs were clustered into subsets based on sequence similarity and/or non-random distribution in relation to the TSS. Non-random distribution of motifs in promoter regions was assessed using a Chi-square test of goodness of fit between the observed distribution and a uniform distribution across the promoter. Motifs defined using YMF/Find Explanators were verified by comparison with motifs identified using MEME [45, 46], repeated 5 times for motif lengths of 6, 7, 8, 9 and 10 bp.

In order to assess the conservation of motifs between *A. nidulans* and *A. fumigatus*, putative upstream regions (300 bp upstream of the translation start codon ATG) were defined for all genes of both species. Motifs were identified in these regions by exact pattern matching using custom PERL scripts. A list of 5872 1:1 orthologue pairs was defined using search options in FungiDB [10]. All gene pairs in this list were scored as having the motif in both species, in *A. nidulans* only, in *A. fumigatus* only or in neither species. The significance of orthologues sharing the motif was tested using a chi-squared test.

### Mutant strain construction and analysis of meaB antisense transcription

The *meaB*Δ*intron* mutant construct, in which the first intron had been excluded, was formed by fusion PCR [81] utilising a proofreading taq polymerase (KOD). The primers used for the first round of PCR were: meaB_F1 (5′-TCTTCGGCTAGTGTCCGAGT-3′) with ΔintronRev (5′-CCGAGGTCGCATAGATAGGTTTCCTGCCACCCTTCCG-3′) and meaB_R4 (5′-TCGAGTGAGTGAGCATTTGG-3′) with ΔintronFor (5′-GGGTGGCAGGAAACCTATCTATGCGACCTCGGAAGAG-3′). The initial products were combined and fused by PCR using primers meaB_F2 (5′-CTCGTAAAGGAGCTGGGTTG-3′) and meaB_R3 (5′-TGCAGGGATGGAGCTTTAGT-3′). This construct was then knocked in at the *meaB* locus by transformation into a Δ*meaB* strain (Δ*meaB:Af-pyrG, pyrG89, pabaB22 riboB2,* Δ*nkuA:argB (argB2)*) [41]. Transformants were selected for the loss of *AfpyrG*, on the basis of resistance to 5-fluoroorotic acid and fidelity of the mutation was confirmed by PCR and Southern hybridisation. A transformant was then outcrossed and the strain used for mRNA assays had the genotype *meaB*Δ*intron pantoB100*. The ΔRNAi strain used was RTMH.7 39 [43] which had the genotype Δ*rrpB*::*pyrG*; Δ*rrpC*::*metG* Δ*dclB*::*pyrG*; Δ*rsdA*::*pyrG*. The *areA49* strain, which has a loss of function mutation due to an 8-bp deletion beginning in *areA* codon 75 [82], had the genotype *areA49 pabaA1.*

Northern analysis was conducted using total RNA as described previously [41, 83]. The *meaB* antisense was probed using the end labelled primer, meaB-ASprobe (5′-GAACCATAATGACATGGCCATGGACCAGGTCGCCCCCAAGTC-3′). Quantification of *meaB* expression was conducted using a phosphorimager (STORM 860) and based on four independent replicates.

## Electronic supplementary material

Additional file 1: **Additional study information.** Additional text, tables and figures describing whole transcriptome and 5’-end alignments and analyses that were not included in the main text. (PDF 479 KB)

Additional file 2: **Whole transcriptome read counts per locus.** Raw and normalised (RPKM) read counts for all annotated loci (n = 10827). 6 loci (CADANIAG00010797, CADANIAG00010692, CADANIAG00010663, CADANIAG00010810, CADANIAG00010773, CADANIAG00010682) encoding spliceosomal RNAs and small nucleolar RNAs annotated as on the + strand were manually altered to the - strand based on depth of coverage across the loci. (XLSX 3 MB)

Additional file 3: **Transcripts predicted by whole transcriptome RNA-seq alignments.** Text file (in ‘.gtf’ format) containing the locations of putative transcripts predicted from alignment of whole transcriptome RNA-seq libraries of cells grown under different conditions. Instructions to view the data using the integrative genome browser (IGV) software are given in Additional file 1. (ZIP 10 MB)

Additional file 4: **Analysis of novel ncRNA and protein coding genes.** Results of searches of novel transcripts against the Pfam and Rfam databases. Each ID in the column ‘transcript_ID’ represents a transcribed region predicted by the Cufflinks software, based on read coverage and its sequence and length are in columns ‘transcript_sequence’ and ‘transcript_length(nt)’. ‘ORF_ID’ indicates the ID of an open reading frame present in the transcript, ‘translated_ORF_sequence’ is its amino acid sequence, ‘proportion_of_transcript_ORF’ indicates the proportion of the putative transcript that is ORF (a high value suggests a real protein coding gene). The following 15 columns descibe the results of a Pfam search, using the translated ORF as the query (see http://pfam.sanger.ac.uk/ for description). The next 15 columns describe the results of an Rfam search, using the putative transcript as the query (see http://rfam.sanger.ac.uk/ for description). The column ‘Since annotated as..’ records cases where the transcript was annotated in a later version of the annotation than the one analysed (red indicates no subsequent annotation). (XLSX 429 KB)

Additional file 5: **Introns.** Text file (in ‘.bed’ format) containing the locations of Introns identified by alignment of whole transcriptome RNA-seq libraries of cells grown under different conditions. Instructions to view the data using the integrative genome browser (IGV) software are given in Additional file 1. (BED 7 MB)

Additional file 6: **Antisense transcription of annotated loci.** Distribution of antisense reads across annotated loci. Raw read counts are shown for all annotated loci longer than 90 bp (n = 10697). 5 loci (CADANIAG00010797, CADANIAG00010663, CADANIAG00010810, CADANIAG00010773, CADANIAG00010682) encoding spliceosomal RNAs and small nucleolar RNAs annotated as on the + strand were manually altered to the - strand. Loci were scored 5’-biased if >40% of reads mapped to the 5’ third and <10% to the 3’ third, 3’-biased if >40% of reads mapped to the 3’ third and <10% to the 5’ third and middle-biased if >75% of reads mapped to the central third. Note that raw read counts were to left, middle and right thirds irrespective of the orientation of the locus. Therefore, for a locus on the positive strand the left third is the 5’ end, while for a locus on the negative strand the left third is the 3’ end. (XLSX 2 MB)

Additional file 7: **Analysis of uncapped transcripts.** Peaks of read head depth >100 in the 'no treatment' 5’-end library. The peaks should represent the 5′ ends of uncapped transcripts. Many of the coverage peaks identify snoRNAs. (XLSX 22 KB)

Additional file 8: **Putative promoter regions.** Text file (in ‘.gtf’ format) containing the locations of putative promoter regions predicted from alignment of 5’-enriched RNA-seq libraries of cells grown under a single growth condition (nitrate as nitrogen source). Instructions to view the data using the integrative genome browser (IGV) software are given in Additional file 1. (GTF 2 MB)

Additional file 9: **Putative promoter regions of annotated genes.** Genomic locations of genes and their associated transcription start sites (TSS) and putative promoters. TSS are classified as 'tight', 'intermediate' and 'diffuse' by their confidence interval length (CIL) value, the length of the confidence interval around the major peak of read coverage representing the transcription start site (<2nt=’tight’, 2nt < CIL < 4nt=’intermediate’, >4nt=’diffuse’). (XLSX 730 KB)

Additional file 10: **Gene ontology analysis of genes with different promoter types.** Gene ontologies (GO) for processes significantly enriched in gene sets associated with ‘tight’, ‘intermediate’ and/or ‘diffuse’ transcription start sites, according to our classification. For each GO category, the ‘cluster frequency’ (of the particular gene set) and ‘background frequency’ (of all annotated genes in the genome) associated with that GO category are reported. The ‘P-value’, Bonferroni-corrected for multiple testing, and ‘FDR’ (false discovery rate) are also reported, followed by colon-separated lists of the genes associated with the GO category and the GO IDs associated with the genes (which may be for the GO category itself, or a more specific sub-category of the broader GO category). (XLSX 442 KB)

Additional file 11: **Motifs that are significantly enriched (calculated by the YMF software) in promoters upstream of 'tight', 'intermediate' and 'diffuse' TSS.** The table shows the motif, the number of occurrences of the motif in the set of promoter regions and the Z-score, measuring how much more common the motif is than a random sequence drawn from DNA with similar base frequencies. These are shown for each set of promoters associated with a type of TSS: ‘tight’, ‘intermediate’ and ‘diffuse’. To distinguish real ‘diffuse’ TSS from stochastic noise (to enrich for real promoters), only those within 500 bp upstream of annotated genes were used. (XLSX 55 KB)

## Abbreviations

TSS: Transcription start site
NAT: Natural antisense transcript
CIL: Confidence interval length
RH: Read head
RT-PCR: Reverse transcription polymerase chain reaction
TAP: Tobacco acid pyrophosphatase
UTR: Untranslated region.
