# Supplementary material for: Transcriptome analysis of the filamentous fungus Aspergillus nidulans directed to the global identification of promoters
Source: BMC Genomics. 2013 Dec 3;14(1):847. doi: 10.1186/1471-2164-14-847 (PMC4046813; doi:10.1186/1471-2164-14-847)
Supplement: Supplementary file 1 — Additional file 1: Additional study information. Additional text, tables and figures describing whole transcriptome and 5’-end alignments and analyses that were not included in the main text. (PDF 479 KB) [file 12864_2013_5557_MOESM1_ESM.pdf]

Additional information on RNA-seq alignments and analyses

Whole transcriptome sequencing

Information about the five whole transcriptome RNA-seq libraries and alignments is shown in the following table. The last column indicates the proportion of bases in the reference genome covered by one of more read, which ranges from 54% to 71% in each individual library and is 79% across all libraries.

Growth condition	Total reads	Mapped reads (%)[a]	Uniquely mapped reads (%)[a]	Bases covered (%)[b]
Nitrate	60,003,026	9,655,629 (16.1%)	9,394,985 (15.7%)	16,868,002 (56.2%)
Complete	60,257,046	11,664,335 (19.4%)	11,381,280 (18.9%)	17,447,319 (58.1%)
Ammonia	46,929,150	7,524,472 (16.0%)	7,324,694 (15.6%)	16,215,410 (54.0%)
-N, 4h	71,009,476	13,370,736 (18.8%)	13,119,404 (18.5%)	18,173,904 (60.5%)
-N, 72h	65,995,033	19,998,301 (30.3%)	19,829,151 (30.0%)	21,426,968 (71.4%)
All	304,193,731	62,213,473 (20.5%)	61,049,514 (20.1%)	23,821,299 (79.4%)

[a] Percentage of total reads in library.
[b] Number and percentage of bases in the reference genome with one or more read aligned (total reference genome size is 30,018,900 bp).

These alignments were used in conjunction with the genome annotation to count the number of reads aligned across each annotated locus. The total number of loci annotated in the genome was 10,827. The following table shows the number of loci with 1 or more, 10 or more and 100 or more reads aligned (either sense or antisense).

Growth condition	>=1 read, sense	>=1 read, antisense	>10 reads, sense	>10 reads, antisense	>100 reads, sense	>100 reads, antisense
Nitrate	9287 (85.69%)	4668 (43.11%)	7693 (71.05%)	1258 (11.62%)	5609 (51.81%)	225 (2.08%)
Complete	9224 (85.19%)	4489 (41.46%)	7871 (72.70%)	1324 (12.23%)	5883 (54.34%)	257 (2.37%)
Ammonia	9286 (85.77%)	4660 (43.04%)	7632 (70.49%)	1171 (10.82%)	5179 (47.83%)	179 (1.65%)
-N, 4h	9395 (86.77%)	4607 (42.55%)	8048 (74.33%)	1388 (12.82%)	6408 (59.19%)	269 (2.48%)
-N, 72h	9721 (89.78 %)	6060 (55.97%)	8656 (79.95%)	2511 (23.19%)	7285 (67.29%)	655 (6.05%)
All	10185 (94.07%)	7754 (71.62%)	9281 (85.72%)	3785 (35.96%)	8137 (75.15%)	1224 (11.31%)

Alignments were also used to define putative transcribed regions based on coverage of the reference genome. The following box-and-whisker plot shows the size distribution of predicted transcripts based on the whole transcriptome RNA-seq alignment (using the Cufflinks program) alongside the sizes of annotated loci in the CADRE annotation (which mostly represent putative full-length genes). Grey boxes represent the interquartile range of predicted transcript lengths, thick horizontal lines mark the median values and whiskers represent values up to 1.5x the interquartile range from the median (circles represent individual outliers). Widths of the boxes are proportional to the relative number of genes/transcripts in each category, showing that there were more predicted transcripts overlapping annotated genes than not. Cufflinks-predicted transcripts were generally shorter than annotated genes, probably due to low coverage transcripts being predicted to be multiple, short transcripts. In addition, predicted transcripts that did not overlap annotated loci were shorter than those that did.

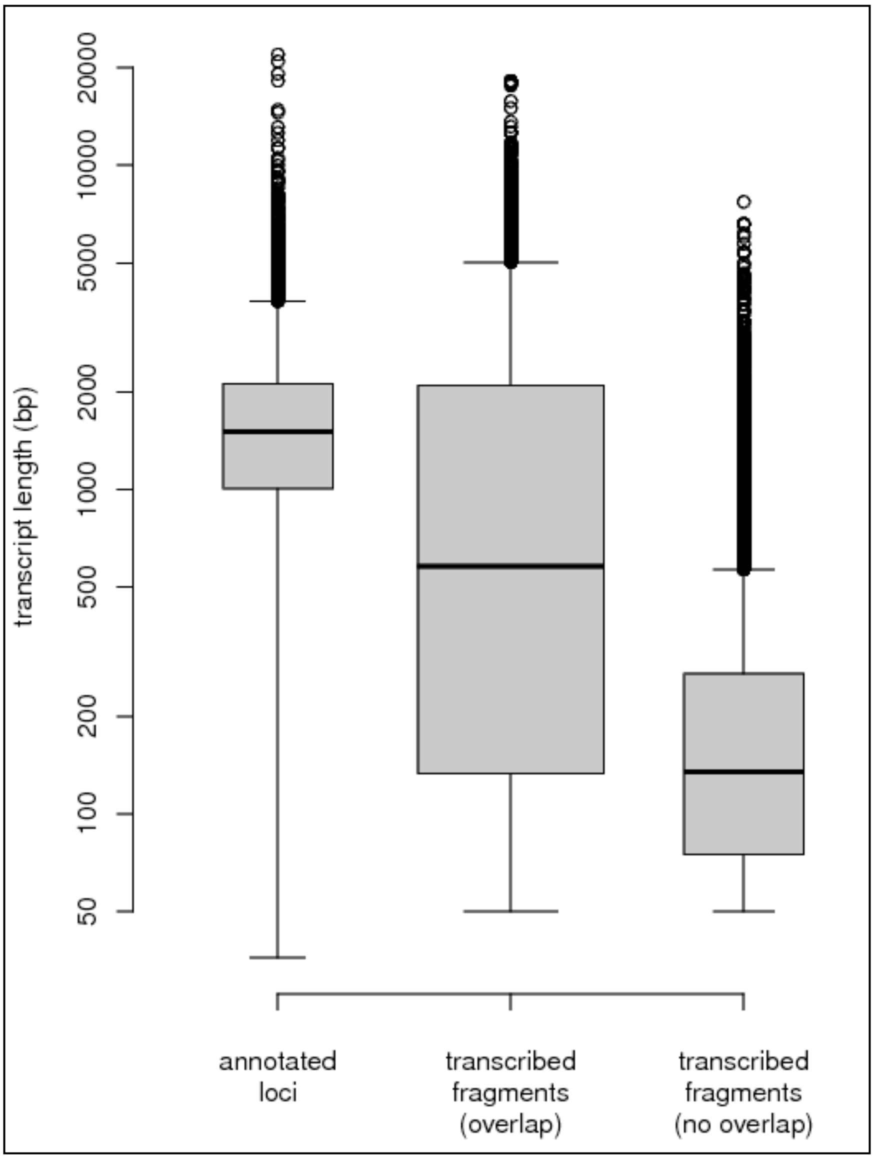

The alignments were also used to identify the locations of introns. A small number of these putative introns appeared to span annotated loci. The following table shows these loci, their locations and the putative introns that span them.

**Locus_ID** [a]	**Description**	**Locus (strand)**	**Intron (strand)**
CADANIAG00010604	8s_rRNA	I:2469191..2469306 (+)	I:2469097..2469418 (+)
CADANIAG00010606	Small nucleolar RNA SNORD14	I:3243757..3243870 (+)	I:3243704..3243938 (+)
CADANIAG00010723	8s_rRNA	I:1693910..1694025 (-)	I:1693807..1694180 (-)<br>I:1693807..1694224 (-)
CADANIAG00004379	conserved hypothetical protein	II:1485521..1486079 (+)	II:1485387..1486285 (-)<br>II:1485429..1486285 (-)
CADANIAG00000550	hypothetical protein	IV:1549955..1551251 (+)	IV:1548800..1551363 (+)
CADANIAG00003514	conserved hypothetical protein	V:2135995..2137053 (-)	V:2134776..2137441 (+)
CADANIAG00010692	Small nucleolar RNA snR75	V:1994205..1994289 (+)	V:1993912..1994578 (-)<br>V:1994143..1994323 (-)
CADANIAG00010627	Small nucleolar RNA snR61/Z1/Z11	VII:2991666..2991751 (+)	VII:2991587..2991816 (+)<br>VII:2991631..2991816 (+)<br>VII:2991631..2991822 (+)
CADANIAG00010745	Small nucleolar RNA Z13/snr52	VII:2992419..2992520 (+)	VII:2992358..2992577 (+)
CADANIAG00002264	HMG box protein, putative	VIII:3513836..3515216 (+)	VIII:3512068..3515656 (+)
CADANIAG00010739	Small nucleolar RNA Z13/snr52	VIII:1606567..1606667 (+)	VIII:1606512..1606737 (+)
CADANIAG00010797	Small nucleolar RNA SNORD14	VIII:1906062..1906170 (+)	VIII:1905990..1906222 (-)<br>VIII:1905997..1906222 (-)

[a] Locus IDs are the CADRE locus IDs, AspGD locus IDs are not given as not all are annotated in AspGD.

5'-end sequencing

Five libraries enriched for transcript 5' ends were sequenced. The following table shows mapping statistics for these libraries. The initial 5' library (TAP-1) was prepared by tobacco acid pyrophosphatase (TAP) treatment to decap transcripts (with no prior alkaline phosphatase treatment). All other libraries were sequenced to a lower depth of coverage. TAP-2 was prepared in exactly the same way as TAP-1. TAP-3 was prepared in the same way except for using a shorter internal adaptor for library preparation. For the 'AP+TAP' library, RNA was treated with alkaline phosphatase to prevent adaptor ligation to uncapped 5' ends. For the 'No treatment' library, no TAP-treatment was applied, so that ligation should occur only with uncapped transcripts.

Treatment	Total reads	Uniquely mapped reads (%)[a]	RH per site [b]
TAP-1	64,148,556	18,817,969 (29.3%)	13.43
TAP-2	2,501,830	870,432 (34.8%)	5.87
TAP-3	4,053,500	2,534,010 (62.5%)	11.46
AP+TAP	1,652,088	864,609 (52.3%)	6.8
No treatment	2,711,023	387,290 (14.3%)	2.87

[a] Percentage of total reads in library
[b] The average number of RH positions per site covered by at least 1 RH

The different libraries were compared. The following figure shows plots of correlation of RH coverage depth among libraries. In general, sites with RH coverage show similar depths of coverage among TAP-treated libraries. However, the library that was not treated with TAP (i.e. should not have been enriched for capped transcript ends) showed poorer correlation with TAP-treated libraries.

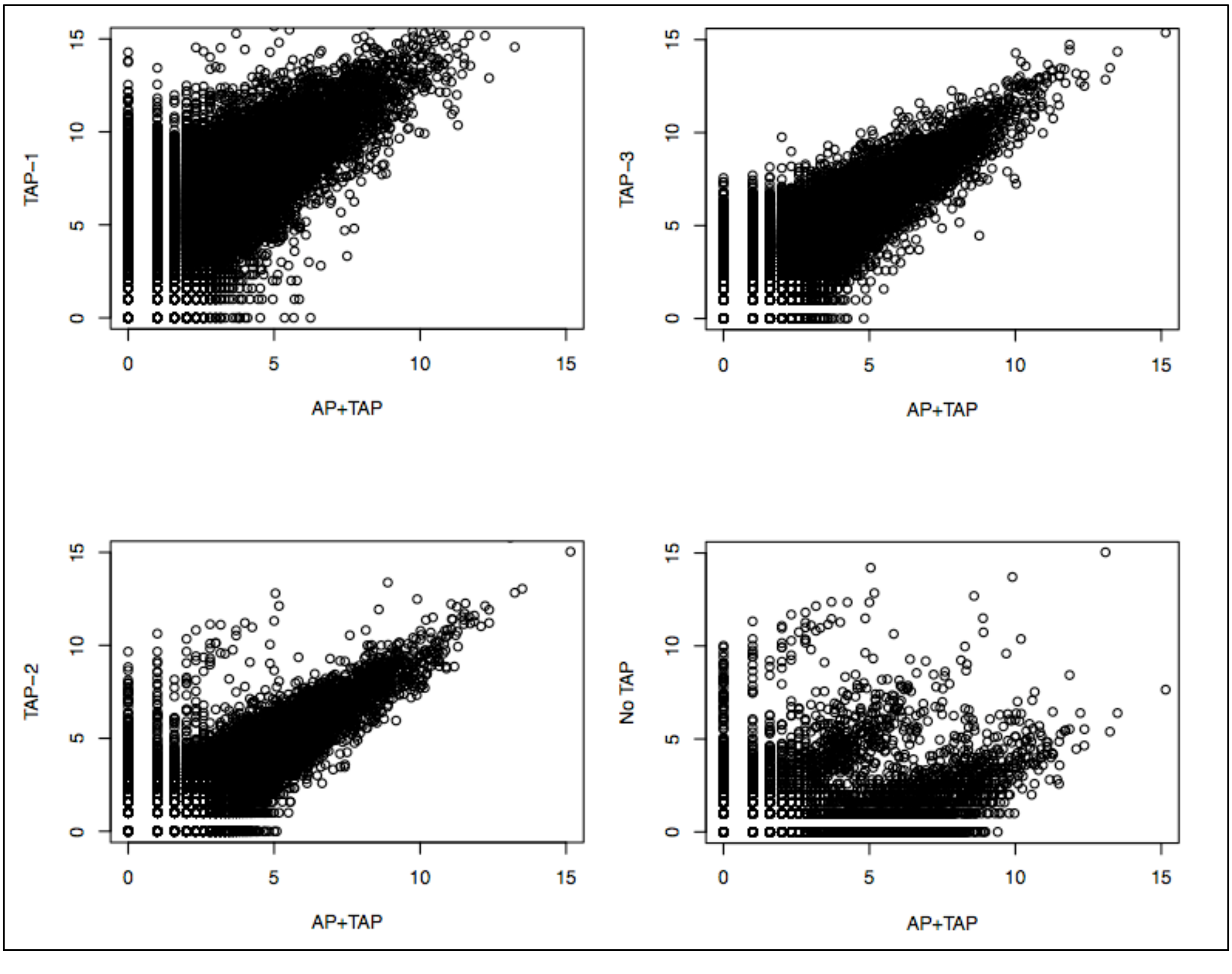

Transcription start sites were defined as ‘tight’, ‘intermediate’ or ‘diffuse’ depending on the distribution of read starts. For each set, the major start site was used to define a putative upstream promoter region of 200 base pairs. Motif enrichment was measured in these promoters and the three most common enriched motifs were CACGTG, CCTNAGG and ACCGCC. The distribution of these motifs was plotted, along with that of known motifs for the TATA box, CAT box, initiator element (InR), heat shock element and AreA- and CreA-binding motifs. Frequency density plots for all motifs in all groups of promoters are shown below.

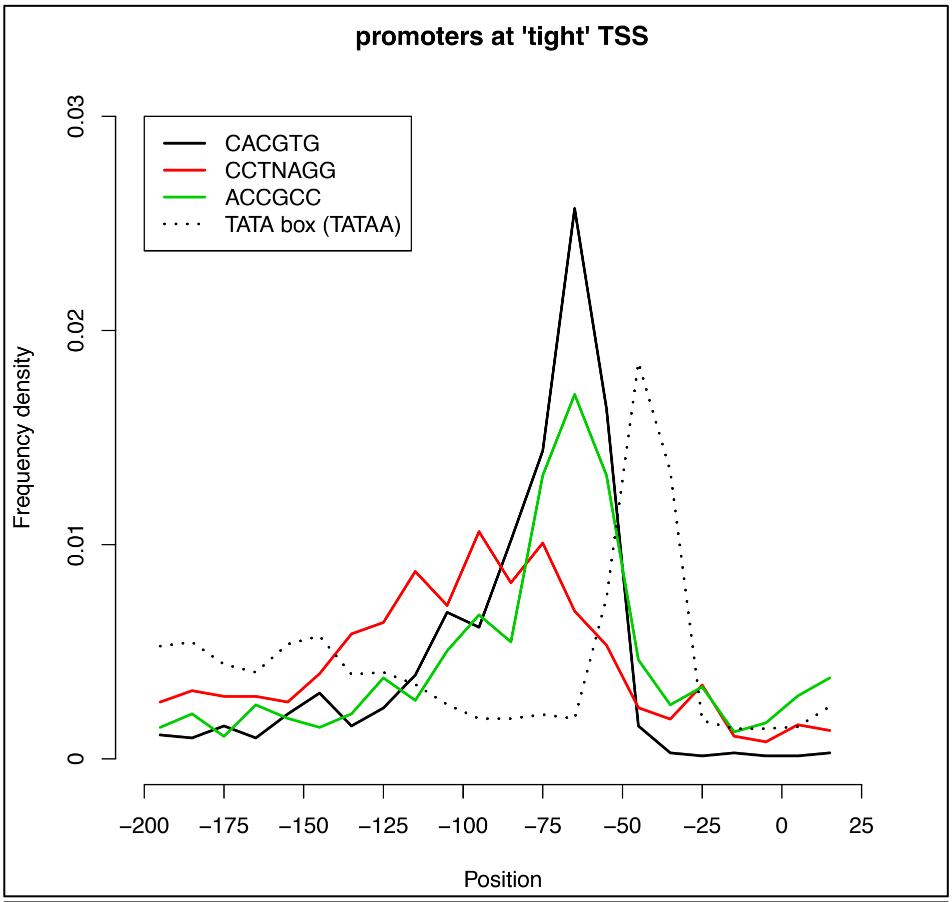

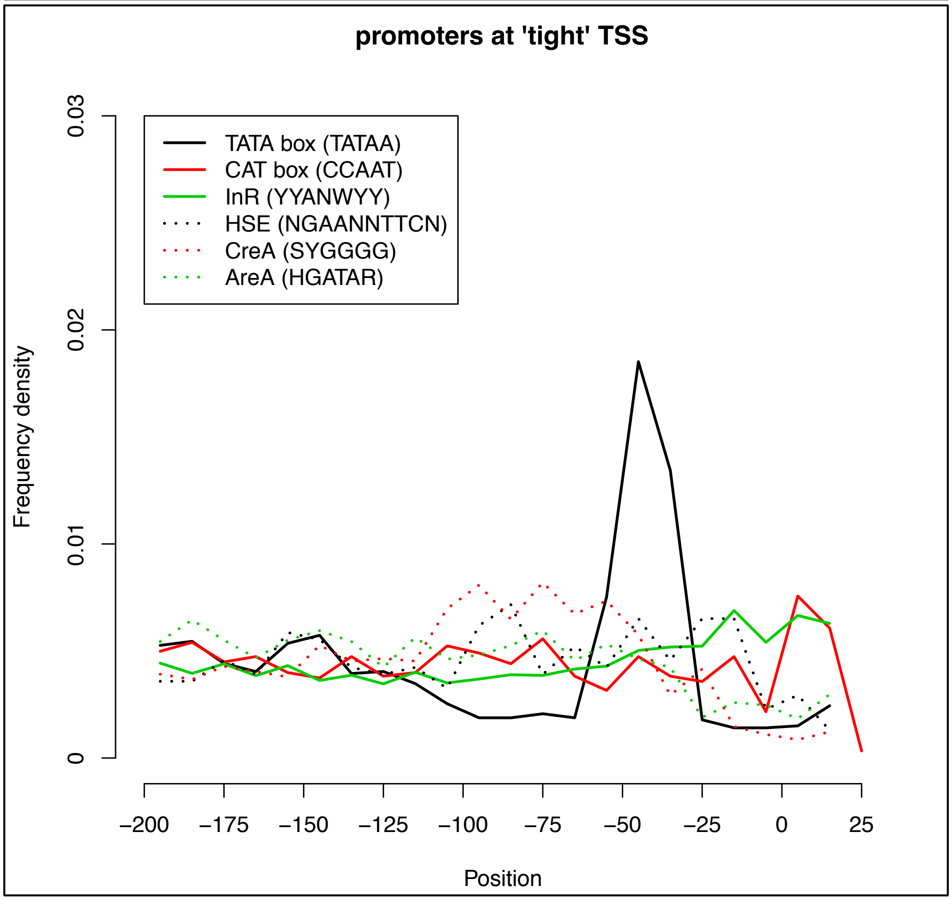

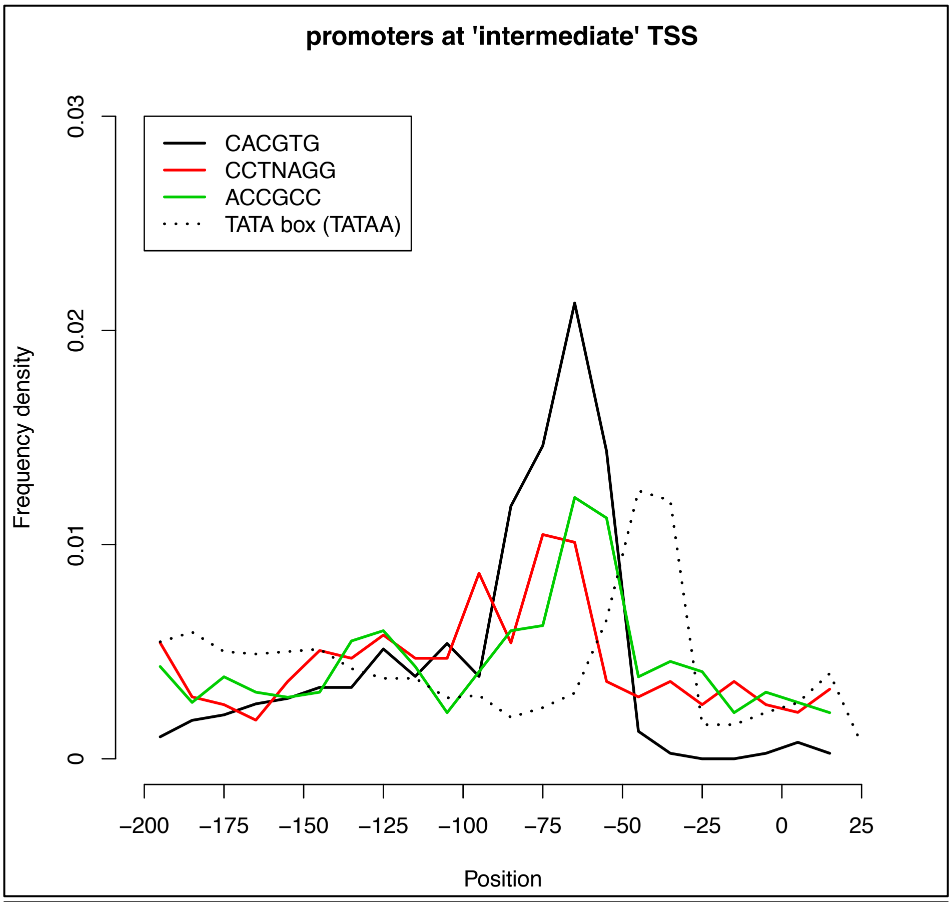
promoters at 'intermediate' TSS
CACGTG
CCTNAGG
ACCGCC
TATA box (TATAA)
Frequency density
0
0.01
0.02
0.03
−200
−175
−150
−125
−100
−75
−50
−25
0
25
Position

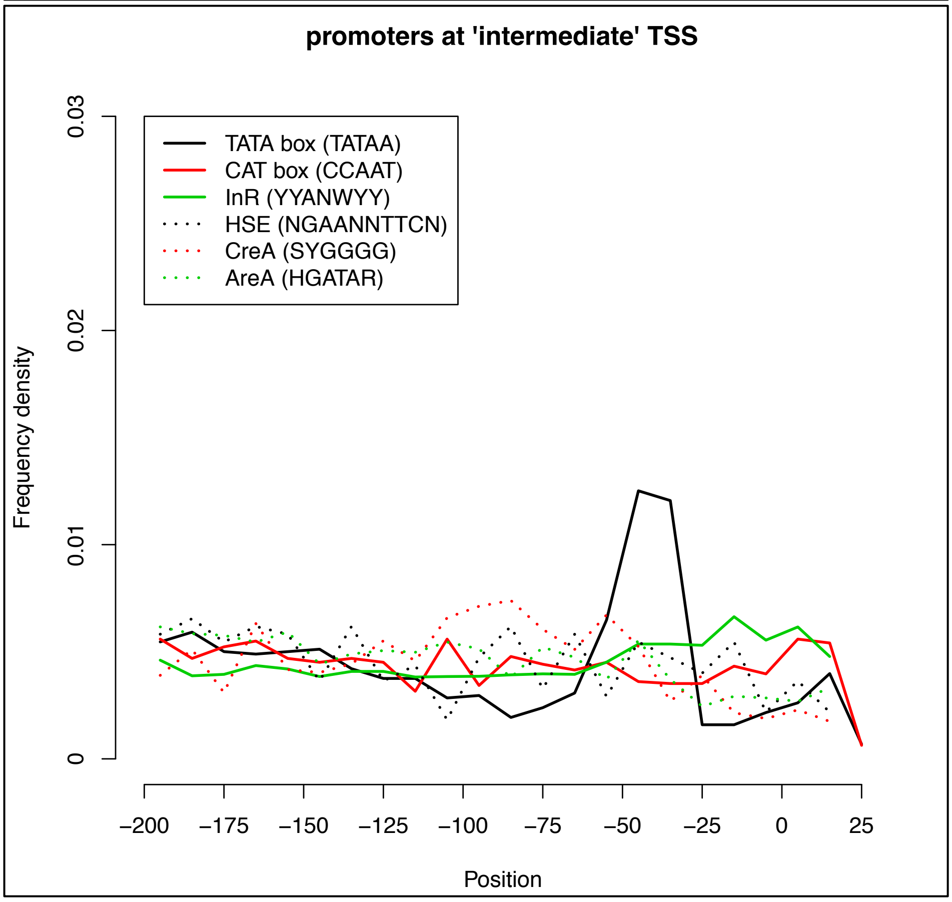
promoters at 'intermediate' TSS
TATA box (TATAA)
CAT box (CCAAT)
InR (YYANWYY)
HSE (NGAANNTTCN)
CreA (SYGGGG)
AreA (HGATAR)
Frequency density
0
0.01
0.02
0.03
−200
−175
−150
−125
−100
−75
−50
−25
0
25
Position

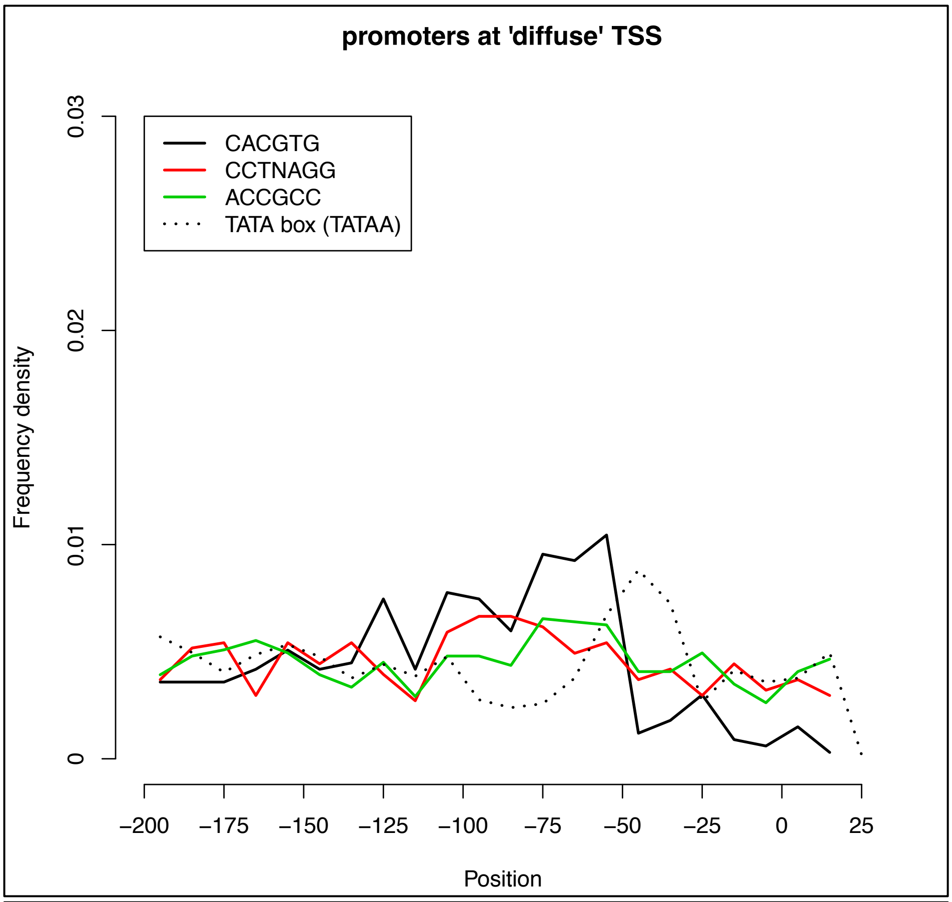
promoters at 'diffuse' TSS
CACGTG
CCTNAGG
ACCGCC
TATA box (TATAA)
Frequency density
0
0.01
0.02
0.03
−200
−175
−150
−125
−100
−75
−50
−25
0
25
Position

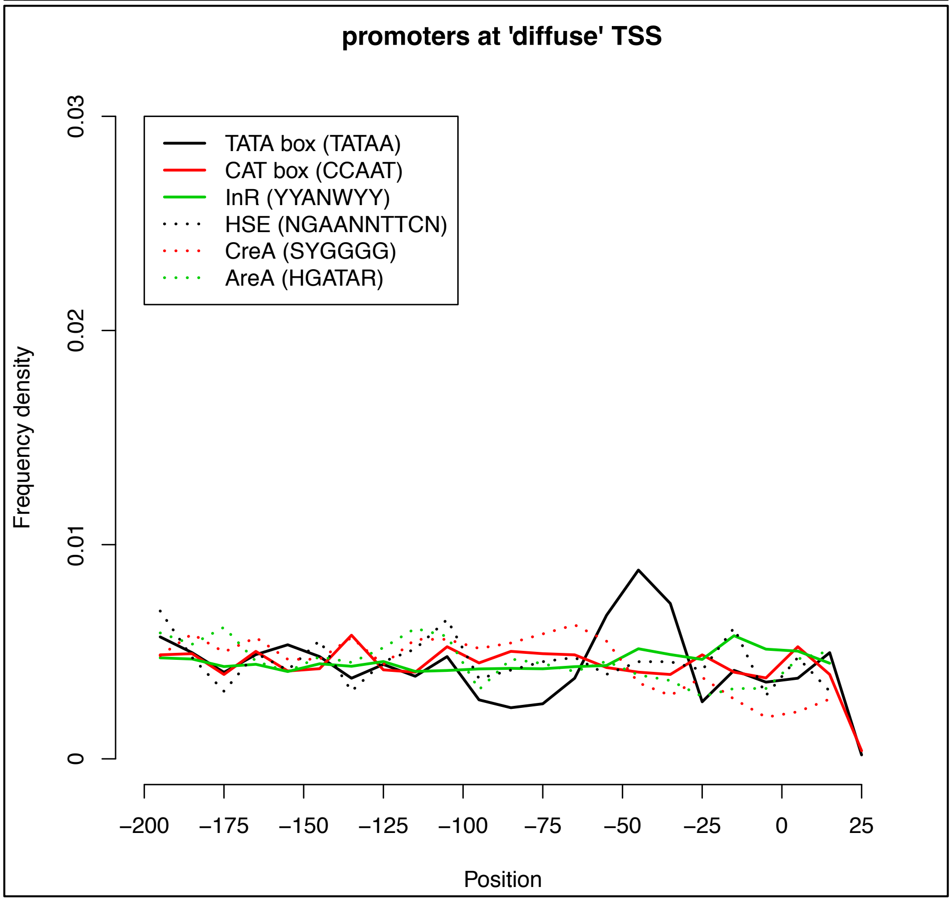
promoters at 'diffuse' TSS
TATA box (TATAA)
CAT box (CCAAT)
InR (YYANWYY)
HSE (NGAANNTTCN)
CreA (SYGGGG)
AreA (HGATAR)
Frequency density
0
0.01
0.02
0.03
−200
−175
−150
−125
−100
−75
−50
−25
0
25
Position

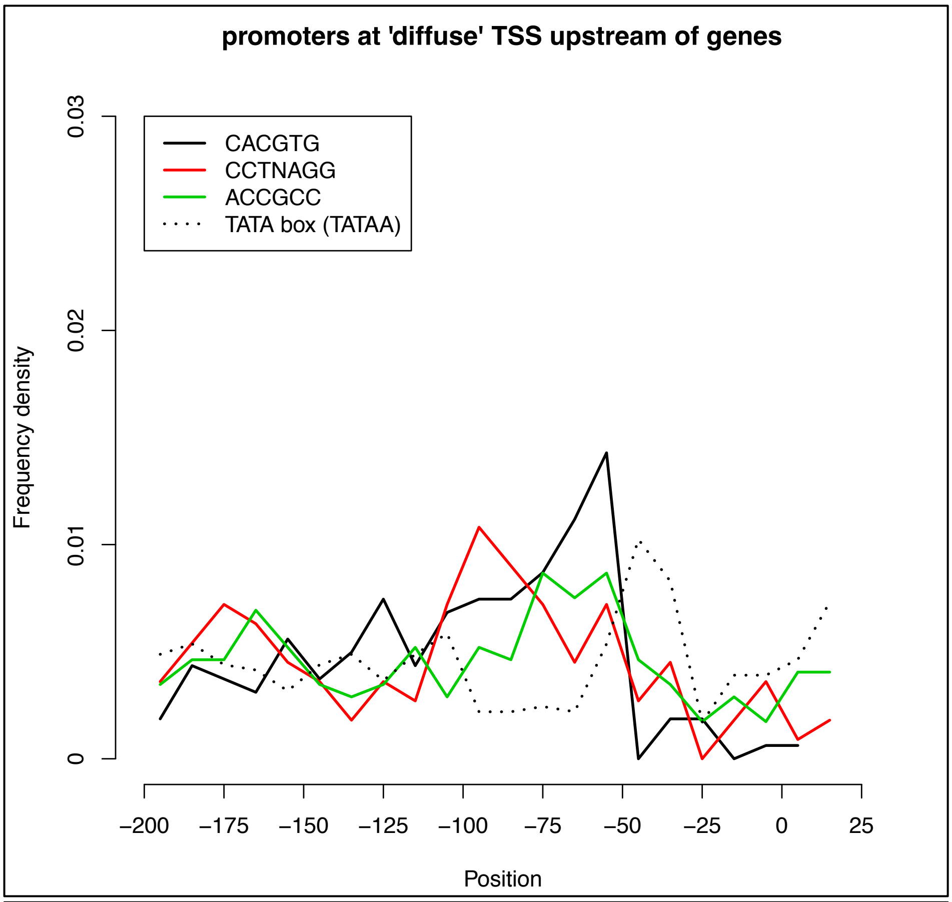
promoters at 'diffuse' TSS upstream of genes
CACGTG
CCTNAGG
ACCGCC
TATA box (TATAA)
Frequency density
0
0.01
0.02
0.03
−200
−175
−150
−125
−100
−75
−50
−25
0
25
Position

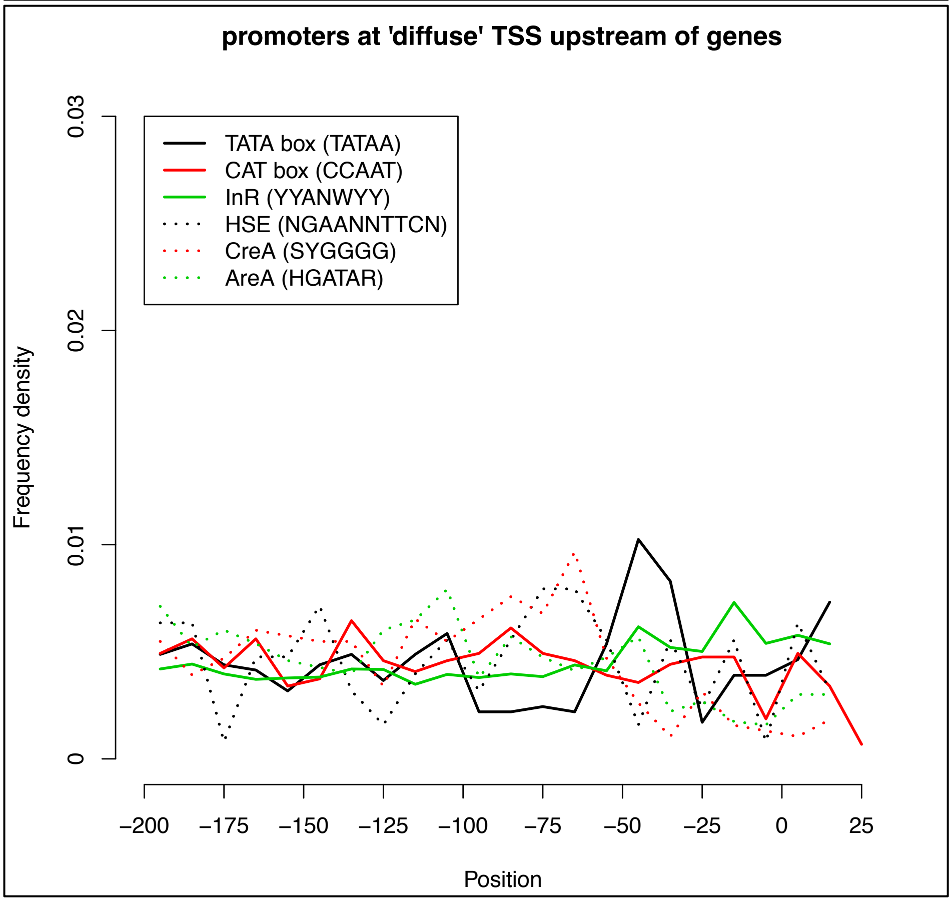
promoters at 'diffuse' TSS upstream of genes
TATA box (TATAA)
CAT box (CCAAT)
InR (YYANWYY)
HSE (NGAANNTTCN)
CreA (SYGGGG)
AreA (HGATAR)
Frequency density
0
0.01
0.02
0.03
−200
−175
−150
−125
−100
−75
−50
−25
0
25
Position

For motifs CACGTG and CCTNAGG (ACCGCC is shown in main text), examples of sequence conservation among Aspergilli are shown. Sequence alignments of regions upstream of the putative translation start codon (ATG; red box) are shown, with conserved motifs boxed in green and the major TSS in *A. nidulans* indicated by a red arrow.

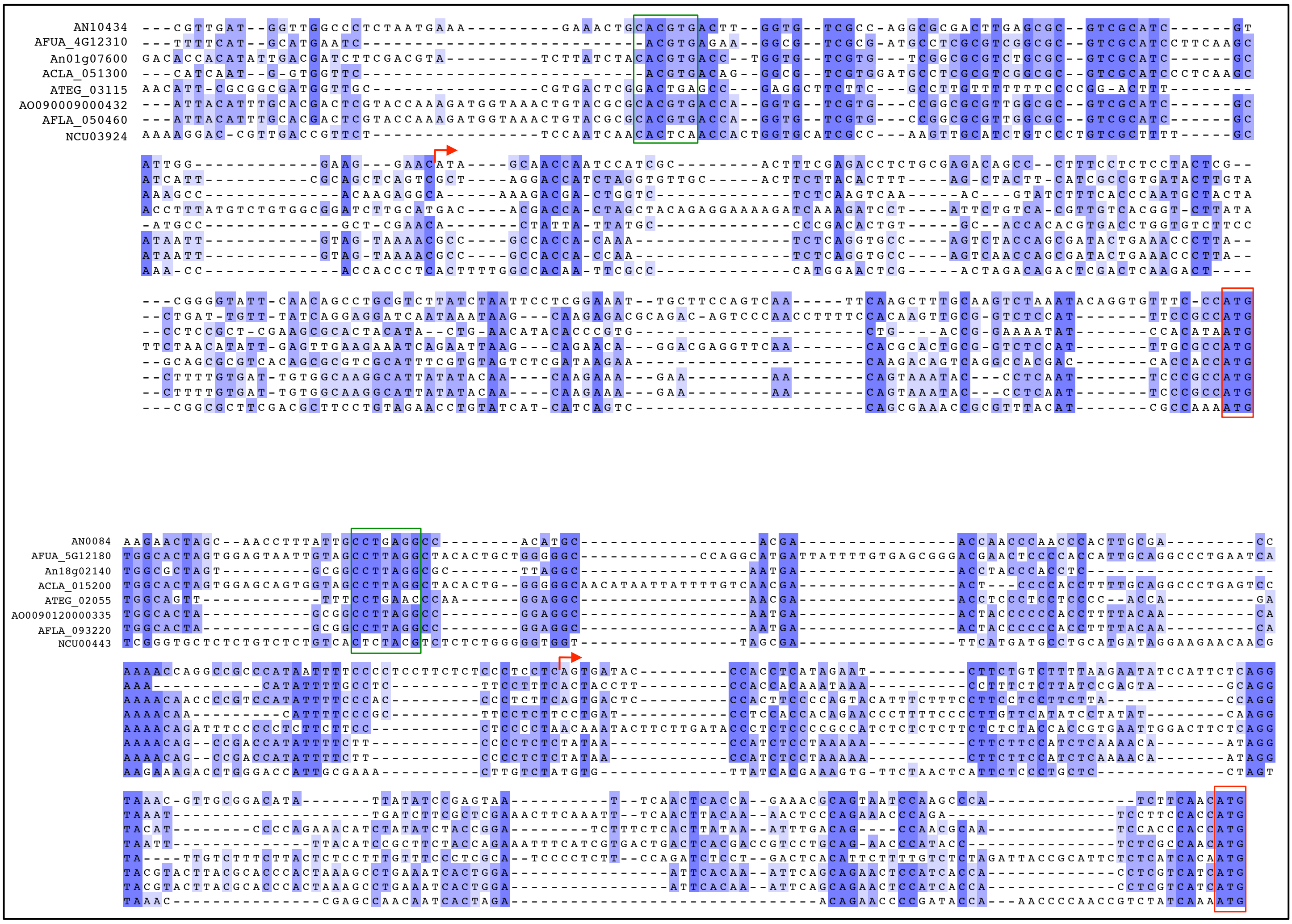

Instructions for viewing the data in Additional files 3, 5 and 8

Additional files 3 and 8 contain data in the "gtf" format. Additional file 5 contains data in the "bed" format. These are specific file formats used to encode genome annotation data. The files are text files, so can be viewed with a text editor, but they are more informative when viewed using dedicated genome browser software. Additional files 3, 5 and 8 can all be viewed using the Integrative Genomics Viewer (IGV). How to do so will be briefly explained here.

IGV can be downloaded from the following location (you will need to register):

```
http://www.broadinstitute.org/software/igv/home
```

It can be installed on computer using the Windows, Mac or unix operting systems, following the instructions on the website. Once installed, IGV requires a reference genome sequence (and an optional genome annotation). The analyses in this study used the CADRE release 5 *Aspergillus nidulans* genome, therefore all genome locations are specific for this genome assembly. Download the CADRE release 5 *A. nidulans* genome sequence and sequence annotation from the following locations:

```
ftp://ftp.ensemblgenomes.org/pub/release-5/fungi/fasta/aspergillus_nidulans/dna/Aspergillus_nidulans.CADRE2.dna.toplevel.fa.gz

ftp://ftp.ensemblgenomes.org/pub/release-5/fungi/gtf/A_nidulans/A_nidulans.CADRE2.5.gtf.gz
```

Once downloaded, uncompress the files. How to do this will depend on whether you are using Windows, Mac or unix operting systems. If using Windows, right-clicking on the compressed file and selecting the option to unzip it usually works. If using Mac or unix, the following commands in the termial should work:

```
gunzip Aspergillus_nidulans.CADRE2.dna.toplevel.fa.gz

gunzip A_nidulans.CADRE2.5.gtf.gz
```

You will need to modify the sequence headers in the "Aspergillus_nidulans.CADRE2.dna.toplevel.fa" file, to remove the initial 3 characters "EG:" after the ">" character (so, for example, ">EG:IV" would be changed to ">IV"). This can be done manually in a text editor (there are 40 sequence headers).

Once this has been done, import the data into IGV as a new genome. Select "Import Genome" from the File menu, then type in a name for the genome reference, select "Aspergillus_nidulans.CADRE2.dna.toplevel.fa" for the "Sequence File" and "A_nidulans.CADRE2.5.gtf" for "Gene File". Save this.

To view the data from this study, import the gtf and bed files (Additional files 3, 5 and 8) by selecting the "Load From File" option from the File menu.

Additional file 3. This is a very large file in gtf format, containing all predicted transcribed regions for each growth condition and all conditions together. Each line of the file contains the label indicating the condition ("nitrate", "ammonia", "complete", "N-starvation_4hrs", "N-starvation_72h" or "all.conditions"). These occur sequentially in the file, so it is possible to split the file and save just the "nitrate" transcripts, for instance, using copy-paste and a text editor. Or, in the terminal of a Mac or unix machine, the following grep command:

```
grep "nitrate." Additional_File_3__all.transcripts.gtf > nitrate_transcripts.gtf
```

Additional file 8. This is also in gtf format. It contains predicted transcription start site regions. Each has a name in the format "TSS_INDEX_CIL", where the INDEX is a unique number used only to differentiate the different TSS and CIL is the confidence interval length used to classify tight, intermediate and diffuse TSS (see manuscript for details).

Additional file 5. This is in bed format, and conatins the intron boundaries identified by mapping the RNAseq reads to the reference for the different conditions ("nitrate", "ammonia", "complete", "N-starvation_4hrs", "N-starvation_72h"). For each feature as viewed in IGV, the thick red parts represent exonic regions bordering the intron and the thin part represents the intron.
